# Multi-omics analysis of SFTS virus infection in *Rhipicephalus microplus* cells reveals antiviral tick factors

**DOI:** 10.1038/s41467-025-59565-w

**Published:** 2025-05-21

**Authors:** Marine J. Petit, Charlotte Flory, Quan Gu, Mazigh Fares, Douglas Lamont, Alan Score, Kelsey Davies, Lesley Bell-Sakyi, Pietro Scaturro, Benjamin Brennan, Alain Kohl

**Affiliations:** 1 MRC-University of Glasgow Centre for Virus Research, Glasgow, UK; 2 Microbes, Infection & Immunity, School of Biosciences, Faculty of Health and Medical Sciences, University of Surrey, Guildford, UK; 3 Leibniz Institute of Virology, Hamburg, Germany; 4 Fingerprints Proteomics Facility, School of Life Science, University of Dundee, Dundee, UK; 5 Department of Infection Biology and Microbiomes, Institute of Infection, Veterinary and Ecological Sciences, University of Liverpool, Liverpool, UK; 6 Departments of Tropical Disease Biology and Vector Biology, Centre for Neglected Tropical Diseases, Liverpool School of Tropical Medicine, Liverpool, UK

**Keywords:** Systems virology, Restriction factors, Virus-host interactions, Proteome informatics, Protein function predictions

## Abstract

The increasing prevalence of tick-borne arboviral infections worldwide necessitates advanced control strategies, particularly those targeting vectors, to mitigate the disease burden. However, the cellular interactions between arboviruses and ticks, especially for negative-strand RNA viruses, remain largely unexplored. Here, we employ a proteomics informed by transcriptomics approach to elucidate the cellular response of the *Rhipicephalus microplus*-derived BME/CTVM6 cell line to severe fever with thrombocytopenia syndrome virus (SFTSV) infection. We generate the de novo transcriptomes and proteomes of SFTSV- and mock-infected tick cells, identifying key host responses and regulatory pathways. Additionally, interactome analysis of the viral nucleoprotein (N) integrated host responses with viral replication and dsRNA-mediated gene silencing screen reveals two anti-SFTSV effectors: the N interacting RNA helicases DHX9 and UPF1. Collectively, our results provide insights into the antiviral responses of *R. microplus* vector cells and highlight critical SFTSV restriction factors, while enriching transcriptomic and proteomic resources for future research.

## Abstract (web summary)

Severe Fever with Thrombocytopenia Syndrome Virus (SFTSV) is a deadly tick-borne virus and a growing global health threat. In this study, Petit et al. used a multi-omics approach on SFTSV-infected tick cells to study its impact and reveal host antiviral responses and key restriction factors.

## Introduction

Severe fever with thrombocytopenia syndrome virus (SFTSV) (*Dabie bandavirus*, *Phenuiviridae, Bunyavirales*) is a tick-borne bunyavirus identified in China in 2009^1^. The human case fatality rate is ~10%, though this may vary by strain^2^, with patients developing a range of symptoms including fever, leukocytopenia, thrombocytopenia, and gastrointestinal symptoms^3^. The virus has been detected in China^1^, South Korea^4^, Japan^5^, Taiwan^6^, and Vietnam^7^, demonstrating a wide geographic distribution. There is an increasing understanding of the human immune responses to infection, immunopathogenesis, and viral counteraction of immune responses (mediated through the activity of the SFTSV non-structural [NSs] protein) during infection, but currently there are no specific antiviral treatments or vaccines available to treat this disease^8–11^.

The genome of SFTSV resembles that of related bunyaviruses, with the L segment encoding the RNA-dependent RNA polymerase (L); the M segment encoding a polyprotein precursor of the viral glycoproteins; and the S segment encoding the nucleoprotein (N) and the NSs protein which is a virulence factor and interferon antagonist in vertebrate infections. The genome termini interact to give a characteristic panhandle structure to the viral ribonucleoprotein complexes, in which the N protein encapsidates the viral genomic or antigenomic sense RNA. Both L and N proteins are critical for viral replication and transcription, however the host factors that regulate these processes during *Phenuivirus* infection in tick cells remain elusive^12,13^.

SFTSV is likely transmitted by several tick species, including *Haemaphysalis (Hae.) longicornis*^14^, *Hae. flava*^15^, and *R. microplus*^6^ to human or other vertebrate hosts. The molecular interactions between ticks and tick-borne arboviruses are poorly understood in particular the interactions of negative-sense RNA viruses, such as bunyaviruses for which most work has been conducted in vertebrate cell lines^16^. The virus-vector interactions are likely to influence virus transmission from the vector to vertebrate hosts, therefore, a deeper understanding is critical from virological, biological, and translational angles.

Tick cells respond to microorganisms and viruses with a range of cellular and immune responses^17,18^, but studies of tick-pathogen interactions at the molecular level are limited, particularly outside the genus *Ixodes*. Published studies have largely focused on tick-borne flaviviruses, such as investigations into the role of RNA interference (RNAi) in arbovirus-tick interactions in *Ixodes* spp.^19–21^. Critically, these studies also described the impact of virus infection on the metabolic, stress, and nucleic acid metabolism pathways within the tick cell^20,22,23^. Recently, the induction of RNAi responses by SFTSV and a potential role for SFTSV NSs as an RNAi suppressor were described during experimental infection of whole *H. longicornis* ticks^24^. That study also demonstrated that SFTSV infection affects metabolic processes, including the Toll pathway, although no functional analysis was performed, showing the considerable gaps in connecting transcriptome data with cellular effects, such as physiological or immunological impacts following infection.

Here we investigated the interaction of SFTSV with cells derived from a natural vector (*R. microplus*) by using a proteomics informed by transcriptomics (PIT) approach to investigate tick protein expression in response to SFTSV infection, exploring the cellular processes beyond the transcriptomic level. We combined the genomic and proteomic information we obtained from SFTSV-infected tick cells to assess the interactome of the SFTSV N protein, and we selected nine targets for further characterization. Notably, two RNA helicase proteins were identified as SFTSV restriction factors: the mRNA decay effector Up-Frameshift 1 (UPF1) protein, a key component of the nonsense-mediated mRNA decay (NMD) pathway^25^; and the multifunctional RNA helicase A DHX9, which plays critical roles in various cellular processes, including the NF-κB antiviral response in vertebrate cells^26^. The identification of these two RNA-binding proteins as SFTSV antagonists represents a significant advancement in our understanding of the molecular mechanisms underlying tick-borne bunyavirus-vector cell interactions.

## Results

### The transcriptome and proteome of *R. microplus* cells

**Fig. 1:**
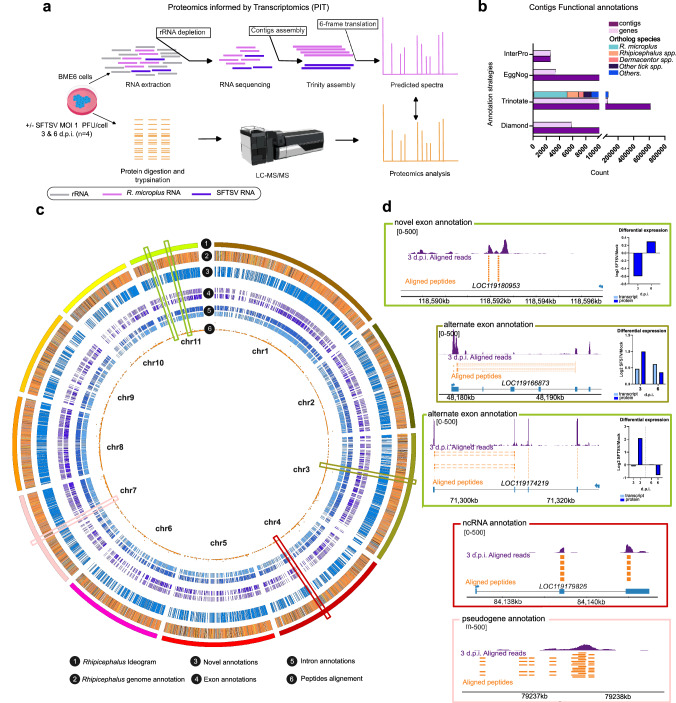
Proteomics informed by transcriptomics (PIT) methodology for *R. microplus* BME6 cells infected with SFTSV. BME6 cells were infected at MOI 1 PFU/cell and samples were collected at 3 and 6 d.p.i. **a** Schematic of the PIT experimental procedure designed for mock and SFTSV-infected BME6 cells (Created in BioRender. Petit, M. (2025) https://BioRender.com/u7ysgw4). **b** Annotation results for the de novo transcriptome; 16,677 contigs (matching to 5813 genes) were identified by Diamond BLASTx search. With Trinotate search we obtain annotation for 70396 gene isoforms. 15,165 contigs (matching to 3427 genes) identified by ortholog search using EggNOG software and 15,165 contigs identified by Interpro protein domain searches, including 2636 complete genes. **c** Circo plot illustrating the Rmic18 genome (GCF _013339725.1_ ASM1333972v1). First band, 1, shows Rmic18 ideogram representing the 11 chromosomes of *R. microplus*. Band 2 shows the repartition of loci on the genome; orange for CDS, blue for gene, pink for pseudogene, green for non-coding RNA, yellow for tRNA, red for CDS with protein, and purple for mRNA. Band 3 represents the 29118 EVM-Pasa annotation generated during our PIT analysis. Band 4 and 5 illustrate annotations at the exon levels (band 4) and the intron levels (band 5). Band 6 represents alignment of peptides against the Rmic18 genome using Exonerate software (see Methods). Scatter plot is used to show the distribution of peptides per loci. **d** Illustrate genome annotation supported by RNA sequencing and proteomics evidence. RNA sequencing and peptides were aligned to Rmic18 genome. Purple color represents RNA reads, orange peptides identified, dashed orange spliced peptides, and blue known gene structures from Rmic18. A bar graph represents the Differential expression of transcripts and protein comparing mock to SFTSV-infected cells (if available). Source data are provided as a Source data file.

Given the limited understanding of tick cell biology, it is of considerable difficulty to study how SFTSV hijacks and re-wires vector cells to establish infection. Therefore, we aimed to establish an optimized study system to investigate SFTSV-tick cell interactions in the *R. microplus* embryonic-derived cell line, BME/CTVM6^27,28^ (hereafter referred to as BME6). In the absence of a well-annotated genome for *R. microplus*, we employed a PIT approach (Fig. 1a), which integrates transcriptomic data to create high-quality protein search databases. This approach is particularly advantageous for non-model organisms with incomplete genomic resources, as it enables the validation of predicted transcripts, identification of previously unannotated open reading frames (ORFs), and characterization of previously unreported splicing events. By combining transcriptomic and proteomic datasets, PIT provides enhanced resolution of cellular pathways and processes, offering insights into tick-specific biology, and characterization of host–pathogens interactions.

In our current PIT approach to generate the mock- or SFTSV-infected BME6 cell transcriptome we collected total RNAs and proteins from SFTSV infected (MOI 1 PFU/ml) BME6 cells (Supplementary Fig.1a–d). A ribosomal RNA (rRNA) depletion protocol adapted from Fauver et al. (2019)^29^ was employed, incorporating customized probes targeting *R. microplus* rRNAs to achieve minimal rRNA detection during sequencing. This strategy facilitated the comprehensive profiling of diverse RNA species, including non-coding RNAs (ncRNAs), viral RNAs, and mRNAs, from both mock- and SFTSV-infected tick cells, thereby enabling an in-depth analysis of transcriptional changes induced by SFTSV infection. This approach, even let us detect tick-specific viral transcripts and proteins, including the complete genome of Wuhan tick virus 2 (*Chuviridae*)^30^ and the IRE/CTVM19-Rhabdovirus^31^, were identified in the BME6 cells used in our study^32,33^ (Supplementary Fig. 1e, f).

Transcripts sequenced from BME6 cells were assembled de novo using sequence reads, from mock and 3 d.p.i. samples, in Trinity^34^ (Supplementary Data 1). The resulting BME6 de novo transcriptome was translated into six ORFs, retaining those longer than 66 amino acids and incorporating all SFTSV proteins listed in the UniProt database, to construct a protein search database for proteomic analysis. Following our initial DIA analysis, we generated a FASTA files containing all predicted ORFs with at least one peptide, constituting a foundational BME6 proteome (17 527 ORFs; Supplementary Data 2). Using this proteome fasta file we identified 5616 unique protein groups, with 4558 of these groups confirmed by the presence of six or more peptides for 4 analyzed samples across all conditions, using the PIT-predicted spectra as the search database for mass-spectrometry analysis (Supplementary Data 3).

To enhance the *R.microplus* genome annotation, the de novo assembled transcriptome and proteome data underwent multiple search strategies including BLAST searches^35^ (Diamond and Trinotate), ortholog searches (EggNOG^36^), and protein domain identification (InterProscan)^37,38^. Among the contigs identified from our BME6 de novo transcriptome, 5813 genes corresponding to 16677 Trinity contigs were identified through Diamond BLASTx searches, and Trinotate (Blastp search) identified 70396 unique gene isoforms from 615521 trinity contigs. Ortholog search performed on uncharacterized contigs with EggNog identified 3427 genes corresponding to 15165 contigs (Fig. 1b, Supplementary Data 4). Ultimately, we were able to identify the protein domain for 2636 distinct trinity contigs using InterProScan’s protein motif search, of which 1437 contigs were previously unannotated (Fig. 1b, Supplementary Data 4). Remaining contigs were classified as transposable elements, non-coding genes or gene loci with no specified function or structure which we designated as uncharacterized and requiring further investigation to establish their function or gene structure (e.g., pseudogenes). Our annotation pipeline resulted in the annotation of 94% of the BME6 proteins identified (Supplementary Fig. 2a).

Finally, we opted to advance our genome annotation efforts by predicting alternative splicing events. To achieve this, we assembled Trinity-derived contigs using the Program to Assemble Spliced Alignments (PASA) software^39^, followed by validation with the EVIdenceModeler (EVM) program^40^, which integrates multiple sources of evidence such as PASA transcripts, protein alignments, and gene prediction tools like *Augustus*^41^. This annotation pipeline enabled the identification of previously uncharacterized BME6 genes and spliced alignments. The 29,118 splicing events identified through PASA-EVM (Fig. 1c, Supplementary Data 5), were grouped on 8000 genes (Supplementary Fig. 2b, Supplementary Data 5). The discovery of gene candidates and splicing events—including the extension of existing ORFs, unreported splicing patterns, and alternative transcript variants (Fig. 1d)—highlights the importance of PIT analysis in studying organisms with limited genome annotations, such as the tick vector *R. microplus*. Moreover, these annotations provided valuable insights into SFTSV infection (Supplementary Fig. 2c), revealing multiple regulated genes with previously uncharacterized features, several of which were validated through both transcriptomic and proteomic evidence (Fig. 1d, Supplementary Fig. 2d). By integrating gene, transcript, and protein data, our PIT approach establishes a comprehensive framework that significantly enhances our understanding of SFTSV infection in vector cells^42^.

### SFTSV infection affects temporal BME6 cellular transcription and protein abundance

**Fig. 2:**
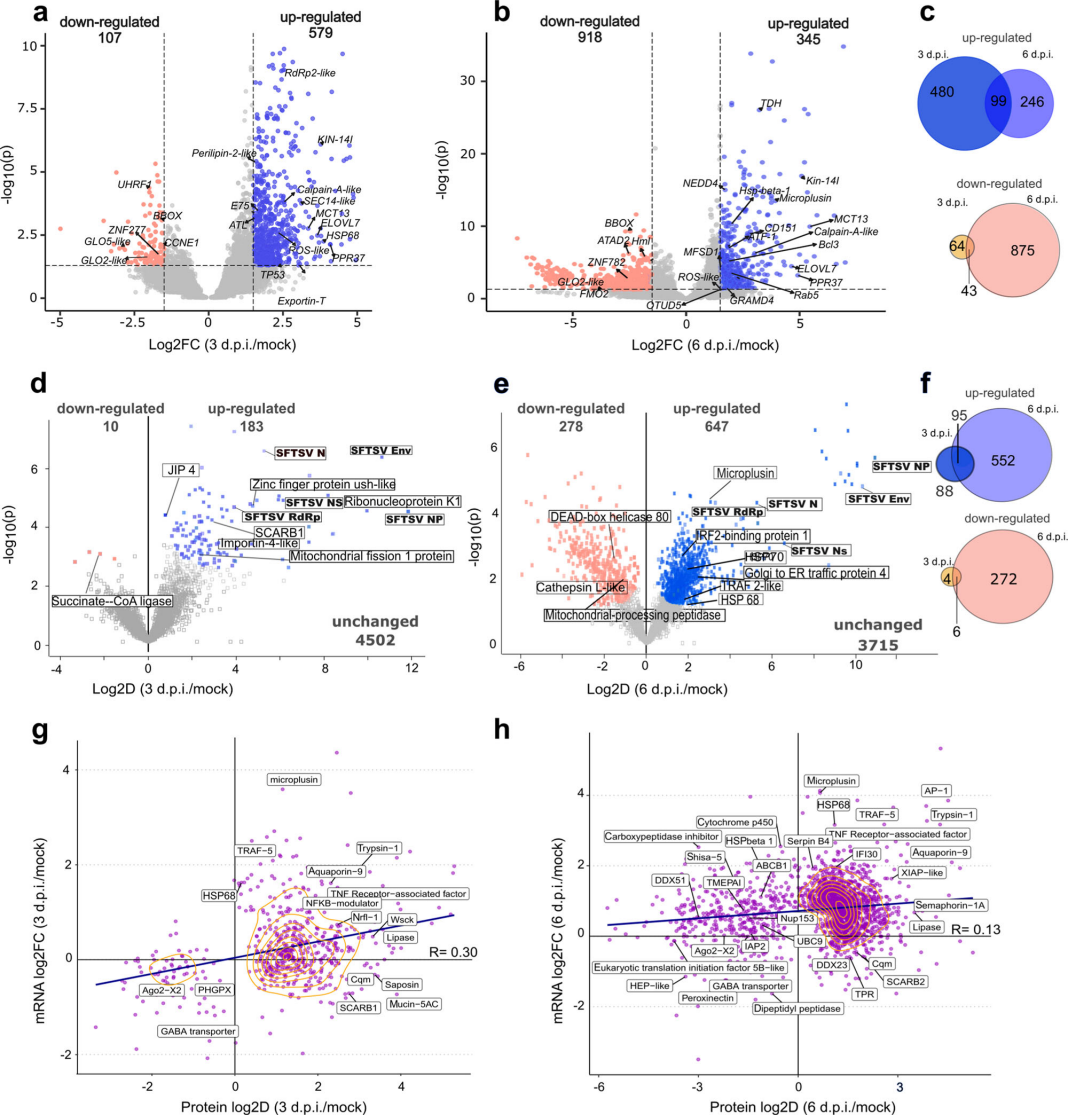
Differential transcript expression and protein levels in SFTSV-infected *R. microplus* BME6 cells. RNA and protein samples were prepared from mock or SFTSV-infected (MOI 1 PFU/cell) BME6 cells and analyzed for mRNA expression and protein biosynthesis in response to infection. **a**, **b** Differential RNA expression volcano plots. X axis represents the Log_2_ Fold Change (Log_2_FC) when comparing 3 d.p.i (**a**) or 6 d.p.i (**b**) to mock-infected cells. The *y* axis shows −Log_10_(padj). Blue dots represent up-regulated transcripts, (Log_2_FC ≥ 1.5, *p*adj ≤ 0.05) and red dots down-regulated transcripts (log2FC ≤ −1.5, *p*adj ≤ 0.05). Gray dots represent non-significantly regulated RNAs (*p*adj ≥ 0.05). Differential gene expression analysis was performed using DESeq2, which models count data from RNA-seq experiments using negative binomial generalized linear models. Genes with an adjusted p value (Benjamini-Hochberg correction) below 0.05 were considered significantly differentially expressed. **c** Venn-diagram representing transcripts significantly differentially regulated (log_2_FC ± 1.5, *p*adj ≤ 0.05) at 3 or 6 d.p.i., blue and red diagrams represent up and down-regulation, respectively. **d**, **e** Differential protein abundance volcano plot. X axis represents the Log_2_ Difference (Log_2_D) when comparing 3 d.p.i. (**d**) or 6 d.p.i. (**e**) to mock infected cells. The *y* axis shows non-zero confidence for each protein (−log_10_(*p*)). Significantly up-regulated proteins (blue dots) and down-regulated proteins (red dots) were identified using a modified *t* test (Perseus “one-sample, two-tailed *t* test” with s₀ = 0.02 and Benjamini–Hochberg FDR ≤ 0.05). Gray dots represent non-significantly regulated proteins. **f** Venn–diagram representing proteins significantly differentially regulated (Log_2_FC ± 1.5, *p*adj ≤ 0.05) at 3 and 6 d.p.i., blue and red diagrams represent up- and down-regulation, respectively. **g**, **h** Scatter plot showing the correlation between protein (*Y* axis) and mRNA (*X* axis) expression ratios, Log_2_D and Log_2_FC, respectively. Purple dots represent genes and/or proteins with *p*adj ≤ 0.5. Density clusters, as defined by R software are represented by yellow lines. **g** Scatter plot showing differential expression of transcripts and proteins at 3 d.p.i. Pearson’s product-moment correlation analysis revealed a moderate positive correlation (*r* = 0.3). A two-tailed *t* test was used to assess whether the correlation differed significantly from zero (*t* = 7.1824, df = 534, *p* = 2.313 × 10^−12^). **h** Scatter plot for differential expression of transcripts and proteins at 6 d.p.i. Pearson’s product-moment correlation analysis revealed a moderate positive correlation (*r* = 0.13). A two-tailed *t* test was used to assess whether the correlation differed significantly from zero(*t* = 9.0447, df = 4370, *p* value < 2.2e-16). Source data are provided as Source Data file.

To elucidate the impact of SFTSV infection on tick cells, we performed a comparative analysis of mock and SFTSV-infected BME6 cells at both the transcriptome and proteome levels. RNA sequencing reads were mapped back to the Trinity-generated de novo transcriptome using STAR aligner^43^. Analysis identified 686 differentially regulated contigs in SFTSV-infected BME6 cells at 3 d.p.i., characterized by a log2FC ≥ 1.5 and a *p*adj ≤ 0.05, compared to mock-infected controls. This difference was even more pronounced at the later time point of 6 d.p.i. (Fig. 2a, b). While 99 up-regulated and 43 down-regulated transcripts were common to both early and late infection time points, the majority of differentially expressed transcripts were unique to each time point (Fig. 2c), suggesting time-dependent viral impact of the cellular RNA pool. We next analyzed BME6 protein alterations using our annotated proteome. We examined proteins that were differentially regulated at either 3 or 6 d.p.i. relative to mock-infected control cells. Our comparison uncovered distinct protein abundance profiles in response to SFTSV infection at 3 and 6 d.p.i. As in our transcriptome analysis, minimal changes were observed at 3 d.p.i. where we identified 10 proteins down-regulated and 183 up-regulated by SFTSV infection (Fig. 2d) compared to a significant shift evident at 6 d.p.i., where 278 proteins were down-regulated, and 647 proteins were up-regulated (Fig. 2e). 95 and 6 proteins were, respectively, significantly up- or down-regulated at both time points (Fig. 2f). Finally, 552 proteins up-regulated and 272 proteins down-regulated proteins were unique to 6 d.p.i., indicating a temporal response to SFTSV. These observations suggest that the timing of SFTSV replication kinetics differently affects the dynamics of protein levels in infected tick cells.

To investigate the effects of SFTSV infection on post-transcriptional regulation in BME6 cells we analyzed the relationship between RNA transcripts levels and protein abundance. The Pearson correlation coefficient was used to evaluate the association between identified proteins with matching transcript. At 3 d.p.i., we observed a modest positive correlation of 0.3 (*p* value = 2.313e-12; Fig. 2g), suggesting a low but nevertheless significant relationship between RNA and protein levels. We found 2 k-means clusters comprising transcript-protein pairs with a strong correlation. This may indicate that various factors influence protein levels beyond transcript abundance. The clustering became more pronounced at 6 d.p.i.; however, the correlation coefficient (*r* = 0.13, *p* value < 2.2.10^−16^) indicates a statistically significant but weak correlation (Fig. 2h). This suggest that SFTSV infection may impact either post-transcriptional, translational processes or both in BME6 cells. Most antiviral genes, identified in previous studies^19,20,23^, that were significantly up- or down-regulated in our transcriptomics and proteomics analysis (*Hsp68*, *Trypsin-1*, *XIAP-like*, *Microplusin*) were excluded from the main density contours (Fig. 2g, h), pointing to distinct regulatory mechanisms for those genes. Altogether our results indicated a complex regulatory landscape governing gene expression and protein biosynthesis in tick cells when infected with SFTSV.

### SFTSV infection regulates BME6 cell immune- and stress-related pathways

To further characterize biological processes altered during SFTSV infection, we ran a pathway analysis, using the recently described *R. microplus* ensemble metazoa gene ontology (GO) categories^42^. Subsequent GO analysis of annotated transcripts and proteins revealed limited alterations at 3 d.p.i. (Supplementary Fig. 3a, b) but significant up-regulation of various GO categories at 6 d.p.i. (Supplementary Fig. 3c, d). For both time points we observed SFTSV-mediated down-regulation of oxidoreductase activity and protein binding categories (i.e., HSP70-interacting protein, NF-κB modulator); and the up-regulation of RNA-dependent RNA polymerase activity, a category associated with the synthesis of small and non-coding RNA in ticks^44^. However, most enriched GO categories were time-dependent, validating our previous observation at the transcriptome, and proteome levels. Although pathways linked to innate immunity or antiviral responses were not identified, the possibility that insufficient genome annotations and GO categorizations^45,46^ contributed to this finding cannot be dismissed.

**Fig. 3:**
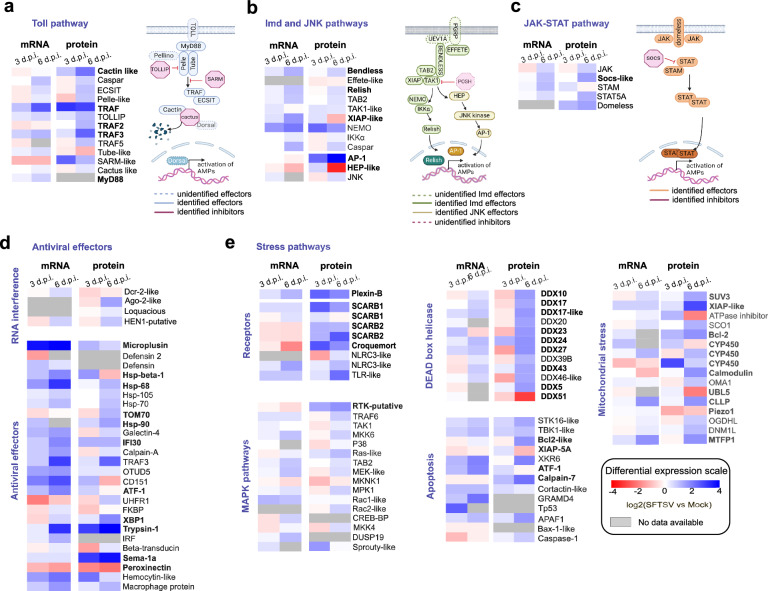
SFTSV differentially regulates core immune and stress pathways in infected *R. microplus* BME6 cells. **a**–**c** Heat map showing the log_2_Fold Change (log_2_FC) of transcripts and log_2_ Difference (log_2_D) of proteins from core immune pathways Toll (**a**), Imd, JNK (**b**) and JAK-STAT (**c**) of BME6 cells infected with SFTSV. mRNA and protein expression were normalized to mock-infected BME6 cells. Associated with their schematic representation, blue in Toll pathway (**a**), green Imd (**b**), yellow JNK (**b**), and orange JAK-STAT (**c**) (Created in BioRender. Petit, M. (2025) https://BioRender.com/7s5zb8h). Dashed line represents a missing protein. **d**, **e** Heat map showing the log_2_FC of transcripts and log_2_D of proteins related to antiviral functions (**d**) and stress-related pathways (**e**) in BME6 cells following SFTSV infection. mRNA and protein expression were normalized to mock-infected BME6 cells. For all panels, heat map gene names in bold represent significantly differentially expressed genes and/or proteins, and gray squares represent genes with no differential expression. Source data are provided as Source Data file.

To elucidate the comprehensive antiviral response mechanisms against SFTSV infection and delineate the core immune pathways in BME6 cells, we integrated previously identified tick-derived immune effectors^20,23,47^ with immune effectors identified in our annotated proteome. This combinatorial analysis let us characterize the effectors of the Toll, IMD, JNK, and JAK-STAT pathways in BME6 cells (Fig. 3a–c), and we observed a moderate up-regulation of these signaling cascades at 6 d.p.i. For example, we observed up-regulation of the AP-1 complex transcription factor part of the JNK pathway (log_2_D = 2.3 (*p* value = 0.06) and log_2_D = 4.5 (*p* value = 0.004) at 3 and 6 d.p.i., respectively).

Additional antiviral effectors associated with arthropod antiviral responses were identified including heat shock factors^48^, and tick-specific antimicrobial effectors such as *Microplusin*, which exhibited an increased mRNA expression for both time points (log_2_FC = 3.67 (*p*adj = 2.44E-12) and log_2_FC = 3.93 (*p*adj = 3.24E-14) for 3 and 6 d.p.i., respectively) and increased protein expression at 3 d.p.i. (log_2_D = 1.15; *p* value = 0.06). *Galectin*^49^, another antimicrobial effector showed increased mRNA expression for both time points (log2FC = 1.2 (*p*adj = 0.03), log2FC = 1.8 (*p*adj = 6E-8) for 3 and 6 d.p.i., respectively) during SFTSV infection (Fig. 3d). In contrast, the mRNA for the antimicrobial peptide *Defensin*, a downstream readout of Toll pathway activity^50^, was down-regulated at 3 d.p.i. (log_2_FC = −1.5; *p*adj = 0.03) and was not detected at 6 d.p.i. or in our proteomic analyses.

Additionally, key components of the RNAi pathway, including Dcr2, Ago2, and Loquacious, were identified and expressed at both 3 and 6 d.p.i. However, no significant regulation of these RNAi effectors was observed during SFTSV infection at 3 or 6 d.p.i., except for Ago2 protein expression, which showed up-regulation at 6 d.p.i. (log_2_D = 1.48 and *p* value = 0.02).

Finally, we examined the regulation of conserved stress pathways, such as the MAPK and apoptosis pathways, which are partly conserved between host and vectors^51^. These pathways have been previously identified as critical for SFTSV infection in human cells, playing key roles in promoting viral replication, immune response modulation, and cell survival during infection^52–54^. We started by examining the regulation of several pattern recognition receptors (PRRs) including the IMD pathway receptor Croquemort^55^ (log_2_D = 2.46; *p* value = 0.00021 and log_2_D = 1.74; *p* value = 0.01, for 3 and 6 d.p.i., respectively) and the Toll-like receptor (TLR-like), which is up-regulated at the protein level at 6 d.p.i. (log2D = 2.27; *p* value = 0.04) (Fig. 3e). We next examined the expression profiles of the MAPK pathways, where all effectors were identified for both time points but only the receptor RTK (log_2_D = 1.42; *p* value = 0.04 and log_2_D = 1.85 *p* value = 0.03; Fig. 3e), and downstream factors Sprouty-like (log_2_D = 1.6 *p* value = 0.005; Fig. 3e), were significantly up-regulated at 3 d.p.i. at the protein level. These data suggest that *R. microplus* MAPK pathways are not involved in the response to SFTSV infection.

As our transcriptomic analysis identified SFTSV regulation of stress-related genes during BME6 cell infection, e.g., 3 d.p.i. up-regulation of *TP53* (log_2_FC = 2.35 *p*adj = 0.04) or 6 d.p.i. up-regulation of *Calpain-A-like* (log_2_FC = 1.43 *p* value = 0.0003) (Fig. 2a), we decided to extend our characterization to examine differential protein expression of the apoptosis and mitochondrial stress pathways. Protein expression of apoptosis effectors, such as APAF1, and XKR6, was up-regulated at 6 d.p.i. during SFTSV infection (respectively, log_2_D = 1.03; *p* value = 0.05; and log_2_D = 1.6; *p*value = 0.01), while expression of apoptosis inhibitors including XIAP or Bcl2-like was unchanged or down-regulated at 6 d.p.i., respectively log_2_D = 0.9 (*p* value = 0.06) and log_2_D = −0.9 (*p* value = 0.01), suggesting the activation of apoptotic processes during SFTSV infection (Fig. 3e). An opposite trend was observed for mitochondrial stress effectors and DEAD box RNA helicases including two anti-bunyaviral candidates identified previously in mammalian cells which were significantly down-regulated at 6 d.p.i., DDX17 (log_2_D = −1.62; *p* value = 0.05) and DDX51^56,57^ (log_2_D = −2.99; *p* value = 0.01) (Fig. 3e). This major down-regulation of DEAD-box helicases at 6 d.p.i. highlights a potential viral strategy to suppress host cellular defences and RNA metabolism. Altogether, our analysis provides a comprehensive examination of temporal proteome and transcriptome level changes in tick cell genes induced by SFTSV infection of *R. microplus*-derived cell cultures (Supplementary Fig. 4).

### SFTSV nucleocapsid protein co-opts stress pathways in both early and late infection

**Fig. 4:**
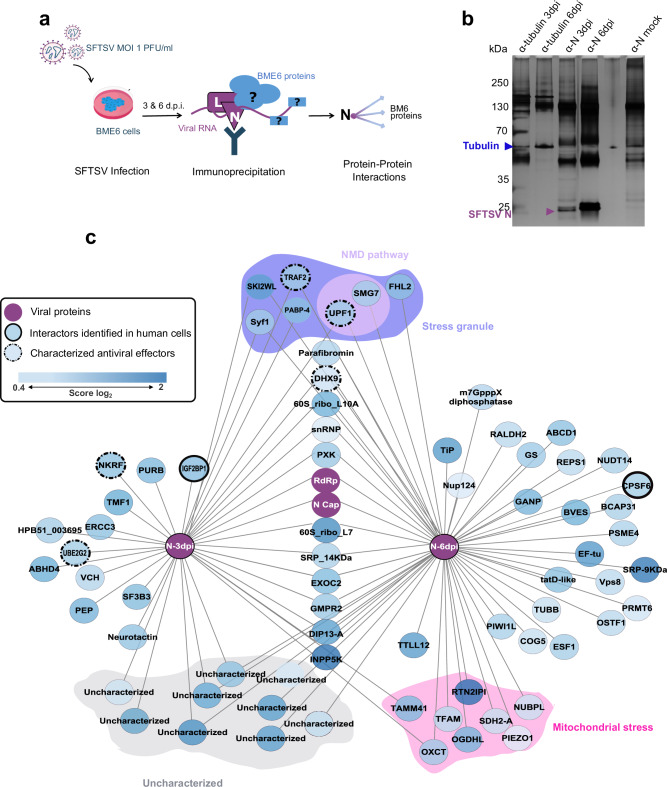
Network representation of the SFTSV N- *R. microplus* interactome in SFTSV-infected BME6 cells. **a** Schematic representation of SFTSV N affinity purification and interactomes identifications (Created in BioRender. Petit, M. (2025) https://BioRender.com/1w1zuzm) **b** Silver staining of mock or SFTSV-infected BME6 cell lysates following immunoprecipitation. Samples were immunoprecipitated with anti-tubulin as control (blue arrow) or SFTSV nucleocapsid protein N (red arrow). **c** SFTSV N protein-protein interaction (PPI) networks at 3 or 6 d.p.i., protein abbreviations/names were determined using the EggNOG ortholog search. Dark purple dots indicate SFTSV proteins, bold black line highlights identified interactors of SFTSV-N protein in human cells^101^, and dashed line represents proteins with previously demonstrated antiviral function. The grey subgroup represents uncharacterized proteins, the purple subgroups represent proteins related to stress granule (purple) and the non-sense mRNA decay pathway (NMD, light purple), while the pink subgroup represents proteins related to mitochondrial stress. Data shown from four independent biological replicates. Significant interactors, all displayed in (**c**), were identified using two-tailed *t* tests combined with permutation-based false discovery rate (FDR) statistics. A total of 250 permutations were performed, with the FDR threshold set at 0.05 to ensure robust statistical significance. The parameter S₀ was set to 1 to effectively distinguish background noise from specifically enriched interactors. The corresponding source data are available in the provided Source Data file.

To further elucidate the intricate interplay between SFTSV and BME6 cells, we investigated the SFTSV N protein interactome with *R. microplus*-derived cells. SFTSV N antibody^58^ was used to immunoprecipitate cellular proteins interacting with the viral nucleocapsid protein N during infection. α-Tubulin immunoprecipitation served as a control. Immunoprecipitated extracts were derived from mock and SFTSV-infected BME6 cell cultures at 3 or 6 d.p.i. and subjected to analysis by mass spectrometry (Fig. 4a). The N interactome data were standardized to control conditions, e.g., anti-N immunoprecipitated lysates from mock-infected cells and anti-tubulin immunoprecipitated lysates from infected cells were examined as a cross comparison to discard non-specific interactors. We identified 16 proteins interacting specifically with SFTSV N at 3 d.p.i., 44 proteins specifically interacting at 6 d.p.i. and 22 common to both time points (Fig. 4c, Supplementary Table 1). Importantly, our data also identified the expected interaction between the viral N and L proteins. We also confirmed interactions of SFTSV N with the cleavage and polyadenylation specificity factor subunit 6 (CPSF6) and the insulin-like growth factor 2 mRNA-binding protein 1 (IGF2BP1) (Fig. 4c) as previously observed in mammalian cell lines^59^. Several of our identified N protein interactors have been previously classified as antiviral effectors, all of which are involved in the NF-κB response to arboviral infection in vertebrate cells, including the Toll receptor-associated factor 2 (TRAF2), NF-κB restriction factor (NκRF), and the DExD/box helicase DHX9^60^. Finally, corroborating our observations on transcriptome and proteome changes, we identified interactions between SFTSV N and key mitochondrial stress factors, as well as proteins associated with stress granules, including an interaction between SFTSV N and Up-frameshift protein 1 (UPF1) which is essential to the NMD pathway^61^ (Fig. 4c). Using a KEGG pathway analysis we were able to assign the remaining SFTSV N interactor to the spliceosome, glycolysis, or mRNA surveillance pathways (Supplementary Fig. 5a). Finally, to understand if uncharacterized proteins were associated with the identified pathways, we performed a protein domain search analysis using InterProScan^38^. Of the nine uncharacterized proteins, seven were associated with functional domains, including tautomerase and transglycosylase domains, RNA recognition motifs, or domains derived from transposable elements (e.g., retrotrans_gag) (Supplementary Fig. 5b, Supplementary Data 6).

### Restriction of SFTSV infection by antiviral, and stress effectors in BME6 cells

While we identified differentially regulated transcripts, proteins, and N protein interactors in SFTSV-infected BME6 cells, this alone does not establish or identify biological significance. To address this limitation, we conducted targeted gene knockdown (KD) experiments in BME6 cells. Leveraging our previous identification of siRNA pathway components in BME6 cells (Fig. 3d, Supplementary Fig. 6a), we employed dsRNA-induced RNAi to silence specific genes of interest.

As transfection of the BME6 cell line had not been previously documented, we identified Magnetofectamine^TM^ O2 (Oz Bioscience) as the best option to deliver target dsRNAs linked to magnetic beads into cells, using a magnetic field (Supplementary Fig. 6b). To evaluate the effectiveness of our approach, we first tested it on SFTSV. We hypothesized that successful dsRNA-mediated knockdown would reduce viral RNA levels, leading to decreased viral mRNA and genome production and consequently viral titers. To assess the efficiency of magnetofection, we utilized dsRNA targeting the SFTSV S segment (Table 1). At 18 h post-dsRNA transfection, BME6 cells were infected with SFTSV at an MOI of 1 PFU/cell. Samples collected at 3 and 6 d.p.i. showed a reduction of SFTSV RNA copy number in infected cells as determined by RT-qPCR of the M segment (Supplementary Fig. 6c) and number of infectious virus particles, assessed by plaque assay (Supplementary Fig. 6d). No changes in cell viability were observed when we compared cells transfected with control dsRNA luciferase or the S segment-dsRNA (Supplementary Fig. 6e). Observed lower level of expression of SFTSV M segment and lower infectivity demonstrated that transfection of dsRNA targeting SFTSV S segment could efficiently knockdown expression of SFTSV mRNA and infectious particles in infected tick cells, validating the use of transfected dsRNA to produce knock-down BME6 cells.

**Fig. 5:**
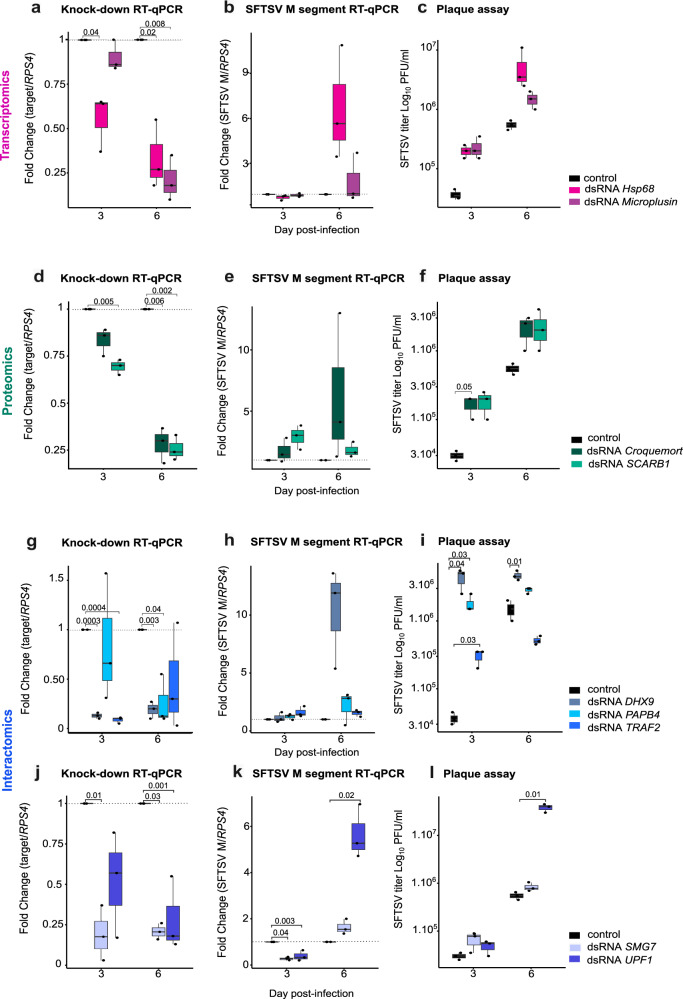
Identification of SFTSV antiviral effectors in *R. microplus* BME6 cells. BME6 cells were transfected with dsRNA targeting specific genes, grouped as follows: **a**–**c** dsRNA against *Hsp68* (*light pink*) and *Microplusin* (*dark pink*). **d**–**f** Proteome-derived targets, including dsRNA against *Croquemort* (*light green*) and *SCARB1* (*dark green*). **g**–**l** Interactome-derived targets, including dsRNA against *DHX9* (*light gray*), *PABP4* (sky *blue*), *TRAF2* (*blue*), *UPF1* (*dark blue*), and *SMG7* (*steel blue*). Panels show: Knockdown efficiency (**a**, **d**, **g**, **j**) measured by RT-qPCR; SFTSV RNA levels (**b**, **e**, **h**, **k**) quantified by RT-qPCR; SFTSV titres (**c**, **f**, **i**, **l**) determined by plaque assay. Data are presented as individual dots representing three independent biological replicates. Box plots display the median (center line), 25th and 75th percentiles (box limits), and whiskers extending to 1.5× the interquartile range. Statistical significance was assessed using paired two-tailed Student’s *t* tests; significant *p* values are indicated where applicable. RT-qPCR fold changes were calculated using the 2^–ΔΔCT^ method with *RPS4* as the housekeeping gene. Source data are provided in the Source Data file.

We next used dsRNA to knock-down selected targets in BME6 cells to identify potential SFTSV restriction factors. Our selection criteria focused on: i) proteins associated with antimicrobial functions; ii) targets significantly up-regulated in our transcriptomics/proteomics analyses and iii) proteins with orthologs identified in *H. longicornis*, the main vector of SFTSV (Supplementary Fig. 7a). These included: *Hsp68* and *Microplusin* from transcriptomic data; *Croquemort* and *SCARB1* from proteomics analysis; and *DHX9*, *PAPB4*, *TRAF2*, *SMG7*, and *UPF1* from our N interactomics dataset. For each target we designed ~500 nt specific dsRNAs (Table 1) that were transfected into BME6 cells at 18 h prior to SFTSV infection. Target silencing was generally effective, though we observed different level of efficacy in our biological replicates. Absence of silencing were observed for *Microplusin*; *Croquemort*; *PAPB4* and *UPF1* at 3 d.p.i. (Fig. 5a, d, g, j). We did not observe any changes in cell viability when comparing to control dsRNA control (Supplementary Fig. 6e).

To assess the impact of target gene silencing on viral RNA levels in SFTSV-infected cells (MOI 0.5 PFU/ml), we quantified the viral M segment by RT-qPCR. Only knockdown of *SMG7* and *UPF1* at 3 d.p.i. resulted in significantly lower M segment levels. These differences were not observed at 6 d.p.i. (Fig. 5b, e, h, k). At this later time point, dsRNA knockdown of *UPF1* resulted in increased viral RNA levels (Fig. 5k). Other genes, including *Hsp68*, *Croquemort*, and *DHX9*, exhibited higher expression of the SFTSV M segment at 6 d.p.i. However, minor variations in values affected statistical significance. These observed variations of fold change at 6 d.p.i. could result of knockdown efficiency variations, or represent the inherent heterogeneity of the BME6 cells, which are derived from embryonic tick tissues^28^.

While RNA quantification can indicate changes in intracellular viral replication levels, it does not necessarily correlate with the production and release of infectious virus particles. Therefore, to obtain a more accurate measure of infectious virus production, we determined viral titre in the supernatant of knockdown (KD) cells by plaque assay (Fig. 5c, f, i, l), thereby measuring the impact of the gene KD on viral infectivity. Silencing of host factors such as *Hsp68* and the antimicrobial peptide *Microplusin* appeared to restrict virus production early on (Fig. 5c), while targeting the N interactor *DHX9*, restricted SFTSV virus production at both 3 and 6 d.p.i., suggesting a critical role for this RNA helicase A during viral infection (Fig. 5i). Finally, we sought to determine if NMD pathway-SFTSV interactions identified from our N interactome studies, impacted SFTSV replication (Fig. 5k, l). *UPF1* and *SMG7*, both NMD effectors and identified as N interactors^61^ (Fig. 4), were successfully knocked down, except for *UPF1* at 3 d.p.i. (Fig. 5j). *SFTSV M segment* RNA levels were unchanged at 3 d.p.i. but increased significantly at day 6 p.i. when *UPF1* transcript expression was ablated (Fig. 5k); this increase was also observed for viral titres, suggesting that UPF1 but not SMG7 restricted SFTSV infection in tick cells (Fig. 5l). Interestingly, *R. microplus* UPF1 is closely related to human UPF1, suggesting potentially similar function(s) in tick and human cells (Supplementary Fig. 7b–d).

## Discussion

Our study provides multi-omics analysis of tick cells infected by a tick-borne bunyavirus. Through PIT analysis of SFTSV-infected *R. microplus* cells, we provide integrated transcriptomic and proteomic profiles of BME6 cells (Fig. 1). Facilitating the characterization of several mRNAs/isoforms, alongside the annotation of 386 previously uncharacterized proteins, our study advances our comprehension of the genomic and proteomic complexity of *R. microplus* BME6 cells. By focusing on the cellular response to SFTSV infection our data demonstrated the conservation and function of various of antiviral mechanisms, including innate immune pathways, stress-related pathways and mRNA surveillance mechanisms (Figs. 2–3). The integration of this improved understanding of tick cell biology with methodologies such as AP-MS and dsRNA knock-down screening has enabled the discovery of viral restriction factors such as the RNA helicases UPF1, and DHX9 (Figs. 4–5). Our findings illustrated the dynamic changes of tick cells biology during viral infection and confirm the sophisticated nature of the arthropod innate immune response to viral infection^62^.

Previous work has used *Ixodes* species to study the antiviral mechanisms present in tick cell lines, but there have been few efforts to use a negative-sense RNA virus-tick model as observed in nature. Only a recent study has used a non-*Ixodes* tick to characterize the RNAi response to viral infection^63^. Here, we developed a *R. microplus* BME6 cell-based model associated with a systems biology approach^64^ to obtain a detailed understanding of SFTSV-tick cell interactions. The generation of a BME6 cell de novotranscriptome and proteome was central to the characterization of these processes. By associating PASA-EVM and ortholog annotations, we joined the collective effort to complete the *R. microplus* genome and proteome to support understanding of the tick-SFTSV co-evolution^17,59^.

Additionally, our rRNA depletion protocol allowed us to identify the regulation of numerous unannotated transcripts, indicative of an expanded ncRNAs repertoire in *R. microplus*. Although the detection of ncRNAs is now standard, their functional roles, particularly in the context of viral infection, are not well characterized^65,66^. For instance, during vertebrate host infections with vector-borne pathogens, ncRNAs have been identified that either indirectly regulate translation via miRNAs or directly through long-ncRNAs^67^. This mechanism could account for the observed low correlation between mRNA and protein levels in our datasets at 6 d.p.i., as shown in Fig. 3. Further research is essential to elucidate the specific roles of ncRNAs in virus-infected tick cells^68,69^.

With our systems virology approach, we improved the characterization of major innate immune pathways of *R. microplus*, including JNK, JAK-STAT, Toll and Imd pathways, which were also identified in *I. scapularis*, showing a high level of conservation across ixodid ticks^42,49^. However, their role in the tick antiviral response remains unclear. While we identified some factors common to tick-borne flavivirus^19,20^ and tick-borne bunyavirus infections, such as the up-regulation of trypsin at days 3 and 6 p.i. and the regulation of complement H factor or SCARB1 (CD36) receptor, better genome annotation and methodologies are still needed to comprehensively identify pan-antiviral effectors across the different virus families^20,23^. Importantly, RNAi serves as a fundamental defence mechanism in arthropods^19^. Our study not only identified RNAi effectors but also leveraged RNAi to facilitate dsRNA-mediated knock-down in tick cells (Supplementary Fig. 6a–c and Fig. 5). Beyond the canonical RNAi components, including Dcr2 and Ago2, our multi-omics analysis also revealed the expression of Dcr1, Ago3 and Aub proteins from the miRNA and piRNA pathways. Further investigations should indicate if, similarly to mosquito cells, tick cells involve for example the piRNA in antiviral responses^70^.

Our datasets, as well as previous studies utilizing flavivirus infections have shown the presence and regulation of various tick cellular stress factors in response to infection^20,21,23,71^. Indeed, flavivirus-infected *I. scapularis* cells demonstrated regulation of apoptosis through regulation of pro-apoptotic factors like Bcl2, and metabolic effectors linked to the stress responses, including oxidative stresses^21^. These processes would appear to confer beneficial impact to the virus, enhancing replication. This shows that both positive and negative-sense RNA viruses can interact with cell stress pathways during tick cell infection.

Importantly, we identified an interaction between mRNA surveillance factor UPF1 and the SFTSV N protein in infected cells. Moreover, restriction of SFTSV replication by UPF1 showed that this RNA helicase regulates SFTSV replication. While we provide the evidence of UPF1 antagonism of a bunyavirus in an animal cell, the mechanism involved remains unclear. UPF1 could degrade viral RNAs via its RNA helicase function conserved in both NMD and the alternative Staufen mRNA decay-mediated (SMD) pathways^61,72^ (Supplementary Fig. 7c,d). Alternatively, the role of UPF1 in stress granules formation may help sequestrate cellular 5′ caps essential for bunyavirus replication, as described for plant-infecting bunyaviruses^73^. The involvement of the NMD pathway in cap-snatching regulation was supported by a second study linking hantavirus N protein, decapping enzyme, and stress granules^74^. This last hypothesis would explain the identification of several proteins related to stress granule formation in our N interactome datasets (Fig. 5c, Supplementary Table 1).

Another RNA helicase, DHX9, displayed a consistent restriction of SFTSV infection in BME6 cells (Fig. 5c, Supplementary Table 1). DHX9 emerges as a multifaceted helicase with pivotal roles in RNA regulation and nucleic acid recognition, exhibiting both pro-viral and anti-viral activities within vertebrate systems^75^. DHX9 is characterized by its dual nucleic acid recognition domains: one domain is specific for DNA, which facilitates pro-viral activities, notably in the context of HIV-1^76^ and herpesviruses^77^ infection. The second recognition domain of DHX9 targets dsRNA and showed anti-viral function against two alphaviruses, chikungunya and Sindbis, for which recruitment of DHX9 to viral replication complexes negatively impacted viral RNA synthesis^78,79^. Although DHX9 has an established antiviral function against DNA viruses in arthropods^80^, our investigation has revealed the restrictive activity of DHX9 against a negative-sense RNA virus in arthropod cells. This finding expands our understanding of the antiviral spectrum of DHX9. However, it also underscores the necessity for additional studies to fully decipher the mechanisms underlying the action of DHX9 in this context.

In conclusion, this in-depth study significantly advances the understanding of tick cell biology and antiviral mechanisms through a comprehensive multi-omics analysis. With the identification of vector cell antiviral restriction factors, UPF1 and DHX9, we demonstrate the suitability of PIT for functional studies of less well-characterized organisms such as the tick arbovirus vector *R. microplus*.

## Methods

### Cells and virus

Cells of the *R. microplus* cell line BME/CTVM6 (BME6), obtained from the Tick Cell Biobank, were grown in L-15 medium supplemented with 20% fetal bovine serum (FBS) and 10% tryptose phosphate broth (TBP) at 28 °C as previously described^28^. Tick cells were grown in 3 ml volumes in sealed, flat-sided tubes (Nunc, Fisher Scientific, UK), with weekly medium changes and subculture at intervals of 2 weeks. African green monkey kidney cells Vero E6 cells were obtained from Michèle Bouloy (Institut Pasteur, France, ATTC CRL-1586) and grown in Dulbecco’s modified Eagle’s medium (DMEM) supplemented with 10% FBS, at 37 °C, in an atmosphere of 5% CO2 in air. The SFTSV strain used in this study was a plaque-purified, cell culture-adapted stock called Hubei 29pp (HB29pp) provided by Amy Lambert (CDC Arbovirus Diseases Branch, Division of Vector-Borne Infectious Diseases, Fort Collins, CO, USA)^81^. Working stocks of SFTSV were generated in the Vero E6 cell line by infecting at a low multiplicity of infection (MOI) and harvesting the cell culture medium 7 days post infection (d.p.i.). Experiments with SFTSV were performed under containment level 3 conditions, approved by the UK Health and Safety Executive.

### Virus infection and plaque assay

BME6 cells were seeded in 12 cm^2^ non-vented flasks with a density of 3$\times$10^6^ cells per flask with 4 ml of L-15 medium. For the PIT experiments, BME6 cells were inoculated with SFTSV at a MOI of 1 PFU/cell, and samples (supernatants and cell lysates) were collected at 3 d.p.i. For the differential gene and protein expression analyses we added a supplementary time point at 6 d.p.i. Regarding biological validation experiments, target KD and parental BME6 cells were infected with SFTSV at a MOI of 0.5 PFU/cell, samples (supernatants and cell lysates) were collected at 3- or 6- d.p.i. For all experiments, virus titres in samples were determined by plaque assay in Vero E6 cells. Briefly, confluent monolayers of Vero E6 cells were infected with serial dilutions of virus made in phosphate buffer saline (PBS) containing 2% FBS and incubated for 1 h at 37 °C, followed by the addition of a Glasgow MEM overlay supplemented with 2% FBS and 0.6% Avicel (FMC Biopolymer). Cells were then incubated for 6 days before fixation (4% formaldehyde) and staining with methylene blue to visualize viral plaques.

### RNA and protein purification from BME6 cells

Approximately 3$\times$10^6^ SFTSV- or mock-treated BME6 cells were scraped into L-15 medium, harvested by centrifugation (100 x g, 10 mins, 4 °C), washed twice with ice-cold PBS and divided into two samples which were then used for either RNA or protein extraction. For RNA isolation, the cell pellet was resuspended in 1 ml of TRIzol® reagent (ThermoFisher Scientific) and purified as described by the manufacturer. For protein isolation, the samples were resuspended in 100 µl of lysis buffer (4% NP-40, 10 mM Tris (2-craboxyethyl) phosphine (TCEP) and 50 mM triethylammonium bicarbonate (TEAB)).

### rRNA depletion, RNA sequencing, and analysis

To perform rRNA depletion of the samples, we modified the Fauver et al. 2019^29^ protocols to fit the peculiarities of *R. microplus* tick ribosomal RNAs. Briefly, we performed nucleic acid extraction using TRIzol® reagent (ThermoFisher Scientific) following the manufacturer’s instructions. The samples were then treated with TURBO DNase (ThermoFisher Scientific) and purified using RNAClean XP beads (Beckman Coulter). For reverse transcription, the RNA was combined with rRNA-specific oligonucleotides (sequences listed in Supplementary Table 2) and dNTPs. Samples were then heat-denatured at 95 °C for 2 min, followed by slow cooling to 50 °C at a rate of 0.1 C/s. Avian myeloblastosis virus reverse transcriptase (AMV, NEB) was added to samples and incubated at 50 °C for 2 h. Subsequently, RNase H (NEB) and DNaseI treatment was introduced to eliminate the RNA from the resultant RNA:cDNA hybrid and the residual oligonucleotides. The resulting RNA was concentrated and purified using the RNA clean & concentrator-5 kit (Zymo Research). Input and rRNA-depleted RNA were analyzed using a 2100 Bioanalyzer (Agilent) per manufacturer’s protocols with the total RNA Pico kit. BME6 rRNA-depleted RNA samples were sent to Azenta Genewiz for a standard RNA-sequencing library preparation (no fragmentation, no enrichment) and sequencing using Illumina NovaSeq 2 × 150 bp sequencing. Following sequence filtering and trimming ~60 million paired end reads were obtained for our 12 samples. The quality of all reads was evaluated using FastQC (v0.11.5, https://www.bioinformatics.babraham.ac.uk/projects/fastqc/). The Trinity de novo assembly software (v2.14.0)^34^, was used to produce a set of assembled transcripts from the RNA-seq data (~2.83 million contigs) using the default parameters.

### Data-independent acquisition (DIA) proteomics

Samples were collected from SFTSV-infected cells at 3 and 6 d.p.i., and mock-infected controls, with four biological replicates analyzed for each condition. Following cell lysis, proteins were resuspended in S-trap binding buffer (90% aqueous methanol containing 100 mM TEAB, pH 7.1) and quantified using the Micro BCA assay. Equal amounts of protein were loaded onto S-trap mini columns, where proteins were captured within submicron pores. The standard S-trap protocol was followed with an increased number of washes (10). Proteins were digested overnight with trypsin, followed by a second 6-hour digestion. Peptides were eluted in 50 mM TEAB, dried, and resuspended in 0.1% formic acid for quantification. Approximately 1.0 µg of peptides were injected into a Thermo Scientific Q Exactive Plus Hybrid Quadrupole-Orbitrap mass spectrometer. DIA was performed using XCalibur software (ThermoFisher). Each DIA cycle consisted of one full MS scan followed by MS/MS scans across predefined isolation windows. The full MS scan was acquired over an m/z range of 345–1155 with a resolution of 70,000 at m/z 200, an AGC target of 3 × 10^6^ charges, and a maximum injection time of 200 ms. MS/MS scans were acquired at a resolution of 17,500 with an AGC target of 3 × 10^6^ charges, a maximum injection time of 55 ms, and a fixed normalized collision energy (NCE) of 25. Isolation windows ranged from 10 to 21 m/z. DIA data were analyzed using Spectronaut software (v16.2.220903.5300) against a custom six-frame translated database generated from transcriptomic data combined with the SFTSV_HB29_Uniprot database. Spectronaut settings included a precursor Q-value cutoff of 0.01 and protein *Q* value cutoffs of 0.01 (experiment-wide) and 0.05 (run-specific). Quantification was performed using QUANT 2.0 (SN Standard), and differential abundance was assessed using unpaired t-tests.

### Proteomics informed by transcriptomics analysis

Files from the DIA MS/MS analysis were first converted to mzML files using Proteowizard^82^ whilst the de novo transcriptome produced by Trinity software (containing ~2.8 M contigs) was translated in to all six ORFs with a start codon and >66 amino acids) using Transdecoder software (Haas, BJ. https://github.com/TransDecoder/TransDecoder) to produce ~6.8 million ORFs. The resultant FASTA files were used in a database search and a subsequent protein annotation pipeline. Carbamidomethyl was chosen as a fixed modification, and N-terminal acetylation, asparagine/glutamine deamidation, methionine oxidation or dioxidation, and conversion of glutamine to pyro-glutamic acid as our variable modifications. Searches were performed with full tryptic digestion and using the following parameters max peptide length 52, min. peptide length 7, missed cleavages 2, m/z max. 1800 min 300. Spectronaut default settings were modified with decoy generation set to inverse; Protein LFQ Method was set to QUANT 2.0 (SN Standard) and data filtering to qvalue; precursor qvalue cutoff and protein Qvalue Cutoff set to 0.01, precursor PEP cutoff set to 0.1 and protein qvalue cutoff (Run) set to 0.05. Major Group Top N, Minor Group Top N and Cross-Run Normalization were not selected. PSM-peptides-proteins FDR of 0.01. This allowed the identification of 12,9200 peptides (unique peptides 77572) corresponding to Trinity-generated transcript ORFs (Supplementary Data 1). The transcripts were mapped to the Trinity files containing all the transcripts using the aligner STAR(v2.7.10a)^43^. The identified transcriptomic and proteomic features were then combined into a single file. In the final step of the workflow, we used diamond BLASTx^83^ to compare each protein sequence against the *R. microplus* genome^42^ (NCBI-RefSeq: GCF_013339725.1) and the NCBI non-redundant (nr) database. To increase the annotation of the remaining non-annotated protein sequences, we used the eggNOG 4.5 algorithm^36^ for ortholog identification and InterProScan 5 search^37^ to identify SUPERFAMILY or Pfam motifs to allow for protein domain annotation. Mock and 3 d.p.i. Samples were used for the PIT analysis. Subsequent analysis added an additional time point 6 d.p.i.

### Gene prediction

Genes were predicted on the RMIC2018 (*R. microplus* 2018*)*, RefSeq genome with the PASA software system^39^ (Program to Assemble Spliced Alignments v2.5.2) in conjunction with the EVM software v2.1.0^40^. We used Trinity de novo assembled transcripts, along with our de novo PIT proteome, as input evidence for the PASA pipeline. Our PASA RNA-seq gene expression results were combined using EVidenceModeler to ab initio gene prediction (Augustus v3.5.0^41^, SNAP v2.0^84^) and protein alignments (exonerate v2.4.0^85^, miniprot v0.12^86^, GenomeThreader^87^—https://genomethreader.org/). We used the following weighted consensus: ab initio prediction assigned value of 1, protein alignment assigned value of 2, Pasa transcript assemblies assigned value of 10. EVidenceModeler software produced a set of 29118 annotations of 8000 genes, all disclosed in our SQLite PASA-EVM database. Virus and virus-like sequences were identified using the taxonomy pipeline (https://github.com/stenglein-lab/taxonomy_pipeline/)^88^.

### Differential gene expression analysis

rRNA-depleted RNA reads were mapped against our de novo transcriptome with STAR (v2.5.2b)^43^. HTseq (v0.6.1) was used to count all reads for each transcripts and set up a read count table^89^. Differential gene expression analyses were performed using the DESeq2 Bioconductor package (v1.30.1)^90^. The default “normal” shrinkage (v1.28.0)^91^ set up was used for analysis. Gene-ontology analysis was performed with the g:Profiler website^92^ using the data Ensembl Metazoa Rmic18^42^.

### Differential protein expression analysis

The Perseus software v.1.6.15.0^93^ was used to further process the DIA proteomics datasets. Protein tables were filtered to eliminate the identifications from the reverse database and common contaminants. In the subsequent MS data analysis, only proteins identified based on at least six peptide and a minimum of three quantitation events in at least one experimental group were considered. The protein intensity values of the interactome dataset were log2-transformed, missing values were filled by imputation with random numbers drawn from a normal distribution calculated for each sample^93^. Results are presented in Volcano plots.

### Affinity purification and mass spectrometry (AP-MS) of SFTSV N protein

BME6 cells were infected with SFTSV HB29 at a MOI of 1 PFU/cell (3$\times$10^6^ cells per 12 cm^2^ flask) and harvested at 3 or 6 d.p.i. by scraping cells into 1 ml of lysis buffer (50 mM Tris (pH 8), 150 mM NaCl, 0.5% NP-40, cOmplete protease inhibitor cocktail, Roche). For each conditions, we analyzed four biological replicates. SFTSV N-affinity purifications (AP) were adapted from AP-MS protocol for Zika virus proteins^94^. In brief, clarified cell lysates were incubated with anti-SFTSV N antibody^81^-coated beads, or Mouse anti-Tubulin antibody (Sigma - T6199) coated beads, at 4 °C, with a ratio 20 µg antibody for 1 mg protein. Non-specifically bound proteins were removed by three washes with lysis buffer and three washes with washing buffer (50 mM Tris (pH 8), 150 mM NaCl). Proteins were eluted in 2% SDS and PBS buffer. Eluted proteins were reduced and alkylated in 10 mM DTT and 55 mM iodoacetamide. The samples were acetone-precipitated twice and afterwards resuspended and denatured in 40 µL U/T buffer (6 M urea/2 M thiourea in 10 mM HEPES, pH 8.0) followed by digestion with 1 µg LysC (FUJIFILM Wako Chemicals) and 1 µg trypsin (Promega) in 40 mM ABC buffer (50 mM NH_4_HCO_3_ in water, pH 8.0) overnight at 25 °C, on a shaker at 800 rpm. Following digestion, peptides were purified on stage tips with 3 layers of C18 Empore filter discs (3 M) as previously described^94^. Samples were analyzed on a nanoElute (plug-in v.1.1.0.27; Bruker) coupled to a trapped ion mobility spectrometry quadrupole time of flight (timsTOF Pro) (Bruker) equipped with a CaptiveSpray source as previously described by Wanner, Andrieux, and colleagues^95^. Raw MS data were processed with the MaxQuant software v.1.6.17.0 using the built-in label-free quantitation algorithm and Andromeda search engine^96^. The search was done against the de novo proteome from PIT analysis in this study (Supplementary Data 2), and the Uniprot entry for SFTSV proteins. The Perseus software v.1.6.15.0 was used to further process the affinity-purification datasets. Protein tables were filtered to eliminate the identifications from the reverse database and common contaminants. In the subsequent MS data analysis, only proteins identified with at least one peptide and a minimum of three quantitation events in at least one experimental group were considered. The iBAQ protein intensity values of the interactome dataset were log2-transformed, missing values were filled by imputation with random numbers drawn from a normal distribution calculated for each sample^93^. Results were represented as a network using Cytoscape^97^. Mass-spectrometry was performed by the Systems Arbovirology team at the Leibniz Institute of Virology, Germany.

### dsRNA production and transfection of *R. microplus* BME6 cells

RNA was extracted from BME6 cells using TRIzol® reagent (ThermoFisher Scientific) and reverse-transcribed using Superscript III Reverse Transcriptase (Invitrogen) following the manufacturer’s instructions. PCR products were generated with T7 RNA polymerase promoter sequences at either end of the fragment using the primers listed in Table 1 and designated as for use in vitro. dsRNA was synthesized using the MEGAscript™ T7 Transcription Kit (Invitrogen) following the manufacturer’s instructions. For control fluorescein (FI)-dsRNA, we replaced dUTP with dUTP-FI. Synthesized dsRNAs were then transfected into BME6 cells to induce KD of the targeted genes. However, as BME6 cells are difficult to transfect by conventional methods, we used a magnetofection (MTX) kit for primary cells (02 Magnetofection, OZ Bioscience). Transfections were carried out in 6-well plates, and 3 μg of dsRNA and 9 μL MTX transfection reagent were allowed to combine with 3 μg of magnetic nanoparticles (Combimag, Oz Biosciences) for 20 min. Nucleic acids were delivered into cells using a magnetic field on the top of the supplied magnetic plate for 30 min. Control for efficient transfection was performed with fluorescein-labeled dsRNA targeting *Renilla* luciferase. Fresh medium was then added to the transfected cells, which were then re-seeded in 12 cm^2^ non-vented flask for subsequent SFTSV infection.

**Table 1:** Oligonucleotide sequences used to generate dsRNA

Gene	Forward (5′-3′)	Reverse (5′−3′)
*Croquemort*	**TAATACGACTCACTATAGGG**CAGCTTGGTCAAGGAGGGAG	**TAATACGACTCACTATAGGG**TTCGAGAAACGTGTAGGGGC
*DHX9*	**TAATACGACTCACTATAGGG**GCAAGTGGCTGTGGACAATG	**TAATACGACTCACTATAGGG**GCTTTAGTAGCCTCCCCACC
*Hsp68*	**TAATACGACTCACTATAGGG**GTGCAAGAGTTCAAGCGGAA	**TAATACGACTCACTATAGGG**CCGTCTCGATGCCTAACGAC
*Humanized Renilla Luciferase* (control)	**TAATACGACTCACTATAGGG**GCGCCCTGGTTCCTGGAAC	**TAATACGACTCACTATAGGG**GAGAATCTCACGCAGGCAGTTC
*Microplusin*	**TAATACGACTCACTATAGGG**CTCACCACTTGGAGCTTTGC	**TAATACGACTCACTATAGGG**CAGCGTTGTGAATCTCCGTG
*PAPB4*	**TAATACGACTCACTATAGGG**AACATCCTGTCTTGTCGCGT	**TAATACGACTCACTATAGGG**ACAAGTTGACGCCCTGGTAG
*SCARB1*	**TAATACGACTCACTATAGGG**GGCATGAACCCAGATCCCAA	**TAATACGACTCACTATAGGG**GTTGCGCACTGCAGTAATCC
*SMG7*	**TAATACGACTCACTATAGGG**AGATTGGGATGTGCAGTGCT	**TAATACGACTCACTATAGGG**CAGAGCATCAGACGAGGGAC
*TRAF2*	**TAATACGACTCACTATAGGG**GACAAGGGCAGTTTCGAGGA	**TAATACGACTCACTATAGGG**GTAGTGTCCGGTCGGGAATG
*UPF1*	**TAATACGACTCACTATAGGG**TCTGCCCAAGCACTTCTCAG	**TAATACGACTCACTATAGGG**AACAGGCACCATACACTCCG
*SFTSV S-segment*	**TAATACGACTCACTATAGGG** GACGCAAAGGAGTGATCATG	**TAATACGACTCACTATAGGG** CAGTTGGAATCAGGGATCC

Oligos were designed based on genomic DNA sequences derived from BME/CTVM6 cells to generate the dsRNA necessary for RNAi-induced gene silencing. Bold sequences represent the T7 RNA polymerase promoter sequence used for in vitro RNA synthesis.

Bases in bold indicate the T7 promoter sequence.

### Cell viability assay

CellTiter-Glo® Cell Viability Assay (Promega) was used to determine the viability of transfected cells. At 3- and 6-days post-transfection, cell supernatants were removed before resuspension of BME6 cells in 200 µl of fresh PBS. 100 µl of the cell resuspension or PBS control was pipetted into opaque-walled 96 well plates in triplicates. The cell resuspension was then mixed with 100 µl of viability reagent, cells were shaken for 3 mins in the dark, and then incubated (again in the dark) for 10 mins at room temperature. Luminescence from each well was measured using the CLARIOstar® Plus plate reader (BMG Labtech) set to detect emission at 545-550 nm.

### Quantitative reverse transcription-PCR (qRT-PCR)

Total RNA of SFTSV-infected and mock-infected BME6 cells was isolated, at 3 and 6 d.p.i., using TRIzol reagent (ThermoFisher Scientific). cDNA was synthesized with SuperScript™ III reverse Transcriptase (Invitrogen). Real-time PCR was performed with iTaq SYBR Green premix (BioRad), and data were collected with QuantStudio 3 RT-PCR system (ThermoFisher Scientific). All Ct values were normalized to the expression values of the house-keeping gene *RPS4*^98,99^ and gene expression quantification was performed by the 2^−ΔΔCt^ method^100^. Oligonucleotide sequences utilized for qRT-PCR are provided in Table 2.

**Table 2:** Oligonucleotides used for RT-qPCR

Gene	Forward (5′−3′)	Reverse (5′−3′)
*Croquemort*	TCATGGCTGGCACGGAAAA	CGGCTGGAAGTAGTATCGCT
*DHX9*	TTTGCCTTACCAGCAGGGAC	TGCAGCCTCGATTTTGCATT
*RPS4*	TCATCCTGCACCGCATCA	ACGCGGCACAGCTTGTACT
*Hsp68*	TCAATCCAGACGAGGCTGTT	GCCGTCTCGATGCCTAACG
*Microplusin*	AAACTGAACTCCAGTGCATCG	ATGACGCAGCTCCTATCCCG
*PABP4*	CGCCCGACCTACAAGTACAC	CCTGGAATGAAGCGGCAATG
*SCARB1*	TCCTACAAAGAGACCAAGCGT	TTCCTTTAGCAAATGCGCCG
*SFTSV genome (M segment)*	AGCCTTCTTCACGACAAGCA	TTCGTCATGGCTCAGGAACC
*SMG7*	CTCGACCTAGTCGCAAGCAT	CGCATATCTCGAGGGTCCTG
*TRAF2*	CAACACTGTTCTCAAGCGGC	TGGAGCATTTGTGTCACCGA
*UPF1*	TGATGAGGAACCCCAGCAAG	GTCTGCGACTCCTTCAGCTT

List of the RT-qPCR oligonucleotides used for the quantification of host gene expression or viral RNA levels in tick cell cultures.

### Immunostaining and confocal microscopy

BME6 cells were seeded onto glass coverslips 24 h prior to fixation. Cells were fixed with 4% paraformaldehyde in PBS for 15 min at room temperature, followed by permeabilization with 0.1% Triton X-100 in PBS for 15 min. Samples were then blocked at room temperature for 1 hour using 4% milk in PBS. Immunostaining was performed with the primary antibody, rabbit anti-SFTSV N^81^, at a dilution of 1:500 for 1 hour at room temperature. Secondary antibody (Goat anti-Rabbit IgG (H + L) Cross-Adsorbed Secondary Antibody, Alexa Fluor™ 568, Invitrogen) was applied at a dilution of 1:1000. Coverslips were mounted with ProLong™ Gold Antifade Mountant with DNA Stain DAPI (Life Technologies) mounting medium, and images were acquired using a Zeiss LSM 710 Meta confocal microscope equipped with a ×40 oil immersion objective.

### *UPF1* phylogeny analysis

The protein sequences of *R. microplus UPF1* gene were compared and aligned with their respective homologs from other representative eukaryote species, including *Drosophila melanogaster*, *Daphnia pulex*, *I. scapularis*, *Apis melifera*, *Bombyx mori*, *Culex pipiens*, *Aedes aegypti*, *Mus musculus*, *Homo sapiens* and *Caenorhabditis elegans*. Multiple sequence alignments and phylogenetic analyses were conducted using MEGA6 software. The neighbor-joining method was employed to construct phylogenetic trees.

### Statistics

For plaque assay of virus replication data, *p* values were calculated using a paired, two-tailed Student’s *t* test. For differential gene and protein expression analysis, significant changes in specific genes or proteins (*p*adj ≤ 0.05) were identified after adjusting for false discovery rate using the Benjamini-Hochberg method. For AP-MS, significant interactors were determined by two-tailed *t* tests with permutation-based false discovery rate statistics. We performed 250 permutations, and the FDR threshold was set at 0.05. The parameter S0 was set to 1 to separate background from specifically enriched interactors. All statistical analyses were performed using R (v4.3.0) or Perseus (v.1.6.15.0)^96^ for proteomics. Cell viability was assessed using a two-way ANOVA test to compare luminescence between day 3 and day 6 and between conditions; no significant differences were observed across all comparisons (GraphPad Prism 10).

### Reporting summary

Further information on research design is available in the Nature Portfolio Reporting Summary linked to this article.

## Supplementary information

Supplementary Information

Description of Additional Supplementary Files

Supplementary Data 1

Supplementary Data 2

Supplementary Data 3

Supplementary Data 4

Supplementary Data 6

Reporting Summary

Transparent Peer Review file

## Source data

Source data

## Data Availability

The mass spectrometry-based proteomics data generated in this study have been deposited in the ProteomeXchange Consortium via the PRIDE partner repository under accession codes PXD054068 (DIA proteomics) and PXD052311 (AP-MS). The transcriptomic data are available in the NCBI database under BioProject accession code PRJNA1116706. Metadata related to RT-qPCR and virus titration experiments are available at 10.15126/surreydata.901607. The Supplementary data 5 (sqlite database for splicing events of BME/CTVM6) generated in this study is available on Figshare at 10.6084/m9.figshare.25637232.v2. Source data are provided with this paper.
