# Supplementary material for: Multi-omics analysis of SFTS virus infection in Rhipicephalus microplus cells reveals antiviral tick factors
Source: Nat Commun. 2025 May 21;16:4732. doi: 10.1038/s41467-025-59565-w (PMC12095547; doi:10.1038/s41467-025-59565-w)
Supplement: Supplementary file 1 — Supplementary Information [file 41467_2025_59565_MOESM1_ESM.pdf]

SUPPLEMENTARY INFORMATION

Multi-omics analysis of SFTS virus infection in *Rhipicephalus microplus* cells reveals antiviral tick factors

Marine J. Petit[1,2*], Charlotte Flory[3], Quan Gu[1], Mazigh Fares[1], Douglas Lamont[4], Alan Score[4], Kelsey Davies[1], Lesley Bell-Sakyi[5], Pietro Scaturro[3], Benjamin Brennan[1*], Alain Kohl[1,6*]

[1]MRC-University of Glasgow Centre for Virus Research, Glasgow, United Kingdom.

[2]Microbes, Infection & Immunity, School of Biosciences, Faculty of Health and Medical Sciences, University of Surrey, Guildford, United Kingdom

[3]Leibniz Institute of Virology, Hamburg, Germany

[4]Fingerprints Proteomics Facility, School of Life Science, University of Dundee, Dundee, United Kingdom.

[5]Department of Infection Biology and Microbiomes, Institute of Infection, Veterinary and Ecological Sciences, University of Liverpool, Liverpool, United Kingdom

[6]Departments of Tropical Disease Biology and Vector Biology, Centre for Neglected Tropical Diseases, Liverpool School of Tropical Medicine, Liverpool, United Kingdom.

*Correspondence: m.petit@surrey.ac.uk; ben.brennan@glasgow.ac.uk; alain.kohl@lstmed.ac.uk

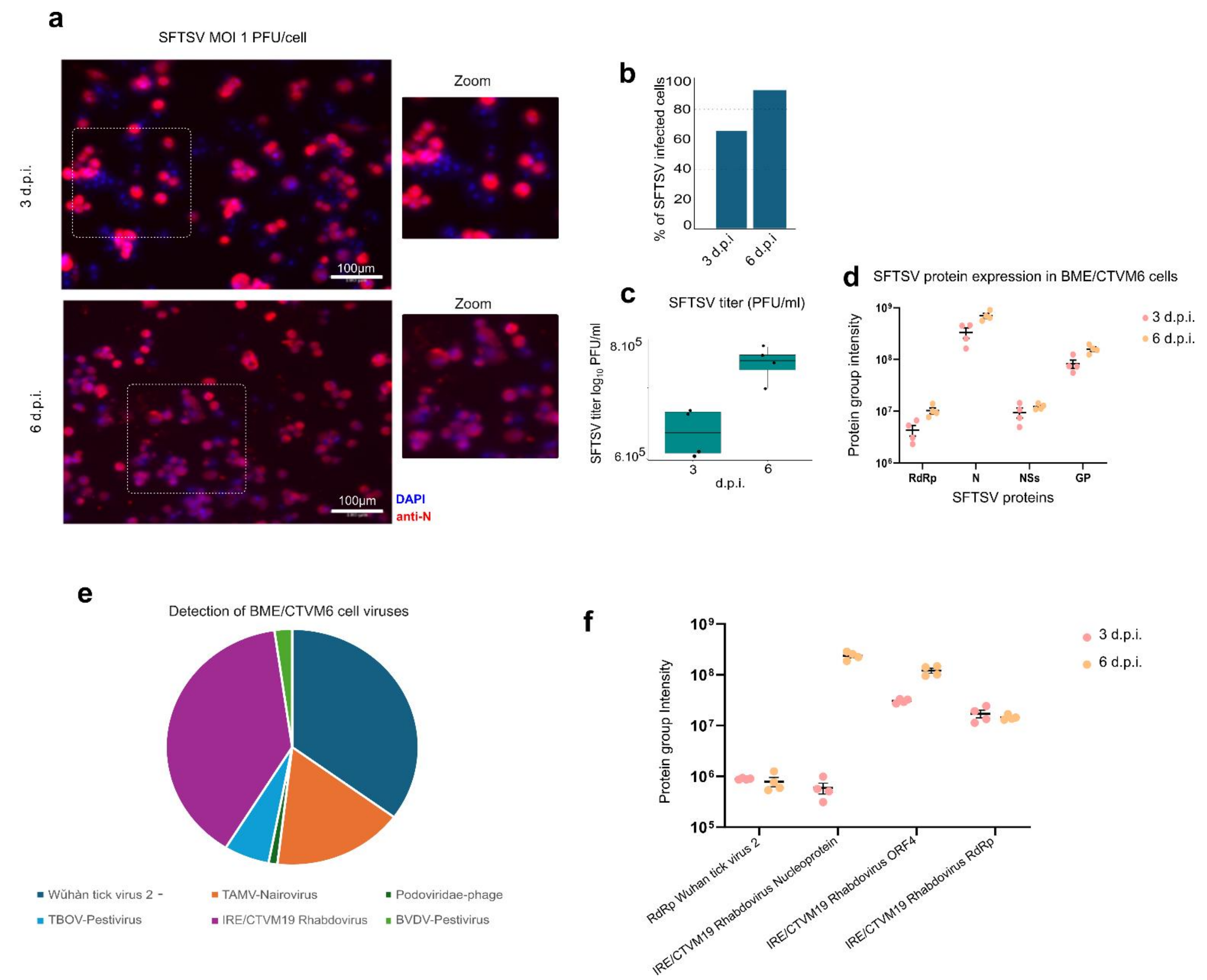

Supplementary Figure 1. BME-CTVM6 cells infection with SFTSV. **a.** Immunofluorescence staining of BME/CTVM6 cells infected with SFTSV (MOI 1 PFU/cell). SFTSV N protein is shown in red (anti-SFTSV-N antibody), and nuclei are stained in blue (Hoechst). Images were taken at 3 and 6 days post-infection (d.p.i.). **b.** Bar graphs showing the percentage of SFTSV N-positive cells (red) relative to total nuclei (blue, DAPI-stained). Cell counts: n = 362 for 3 d.p.i. and n = 448 for 6 d.p.i. **c.** Quantification of SFTSV viral titres by plaque assay, expressed as Log_{10} PFU/mL. Dots represent biological replicates (n = 4). **d**. Protein expression levels of SFTSV proteins as determined by mass spectrometry. Proteins include Nucleoprotein (N), Non-structural protein (NSs), Glycoprotein (GP), and RNA-dependent RNA polymerase (RdRp). Dots indicate protein group intensity for each biological replicate (n = 4) at 3 d.p.i. (pink) and 6 d.p.i. (orange). Data are presented as mean values ± SEM. **e**. Pie chart showing viral RNA reads identified in RNA-seq data. Viral sequences were annotated using the taxonomy pipeline (https://github.com/stenglein-lab/taxonomy_pipeline/), revealing the presence of tick-associated viruses. Wuhan tick virus 2 is shown in dark green; TBOV-Pestivirus in blue; TAMV-Nairovirus in orange; IRE/CTVM19 Rhabdovirus in purple; Podoviridae-phage in green; BVDV-Pestivirus in light green. **f.** Protein expression levels of viruses detected in BME/CTVM6 cells, including Wuhan Tick Virus 2 RdRp and IRE/CTVM19 Rhabdovirus proteins (ORF4, Nucleoprotein, and RdRp). Dots represent protein group intensity for each biological replicate (n = 4) at 3 d.p.i. (pink) and 6 d.p.i. (orange). Data are presented as mean values ± SEM. Source data are provided in the accompanying source data file.

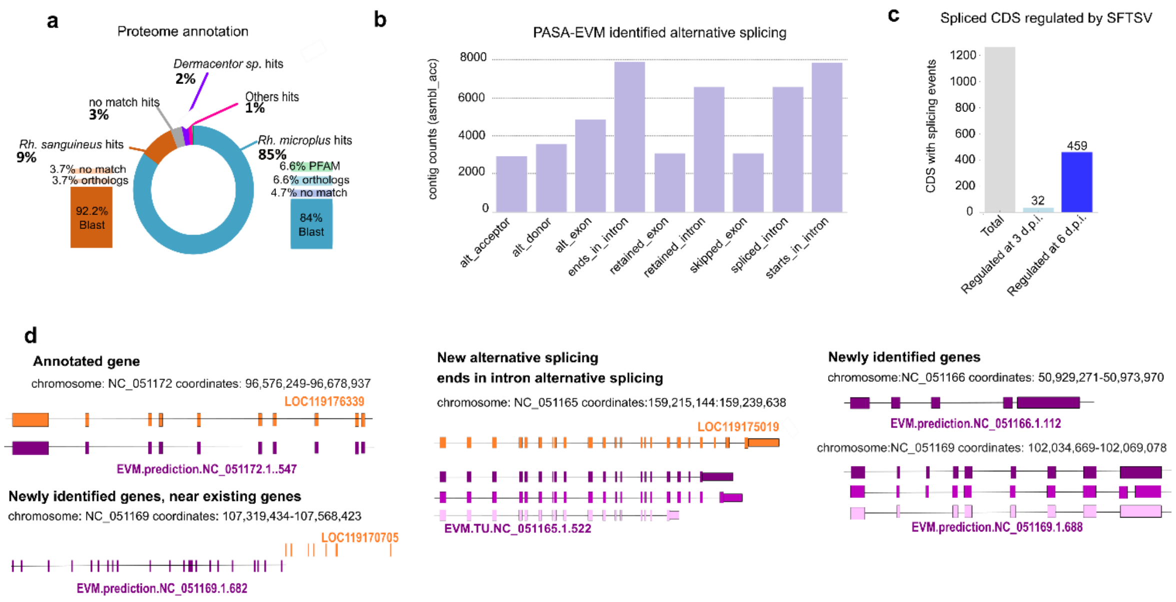

Supplementary Figure 2. Rmic18 novel genomic annotation. a. Annotation results for the BME6 proteome, of 4750 proteins identified by PIT, 85% were identified as *R. microplus* (cyan) by BLAST, orthologs search (EggNOG), and protein domain identification (PFAM) as presented in the bar plot. 9 % were associated with *R. sanguineus* (brown), by BLAST search or ortholog search (EggNOG). Finally, 3% of the proteins did not yield a match (grey), 2% associated with *Dermacentor* species (purple), and 1% with other species (pink). **b.** Gene counts associated with newly identified alternative splicing events identified via the PASA-EVM pipeline. **c.** Bar graph representing identified coding sequence (CDS) annotated with at least 1 new splicing events from all sequences and from significantly SFTSV-regulated CDS. The grey bar represents all identified splicing events. Events occurring in coding sequences (CDS) and regulated by SFTSV at 3 days post-infection (d.p.i.) are shown in light blue, while those regulated at 6 d.p.i. are shown in dark blue. **d.** Examples of novel annotations or novel splicing events in the *R. microplus* genome. Boxes represent introns and lines represent exons. Orange gene structures labeled with a LOC number correspond to VectorBase IDs from the current RMIC18 genome annotation (Ensembl Metazoa). Dark purple gene structures indicate newly identified PASA-EVM annotations, while light purple represents isoforms. EVM IDs were used to reference the database. Selected examples illustrate different annotation types: a gene identical to the reference (positive control), a novel annotation with alternative splicing (e.g., alternative transcript ends), and a completely new gene structure. Source data are provided as Source data file. And Supplementary data collecting the novel annotation is available in Figshare [10.6084/m9.figshare.25637232].

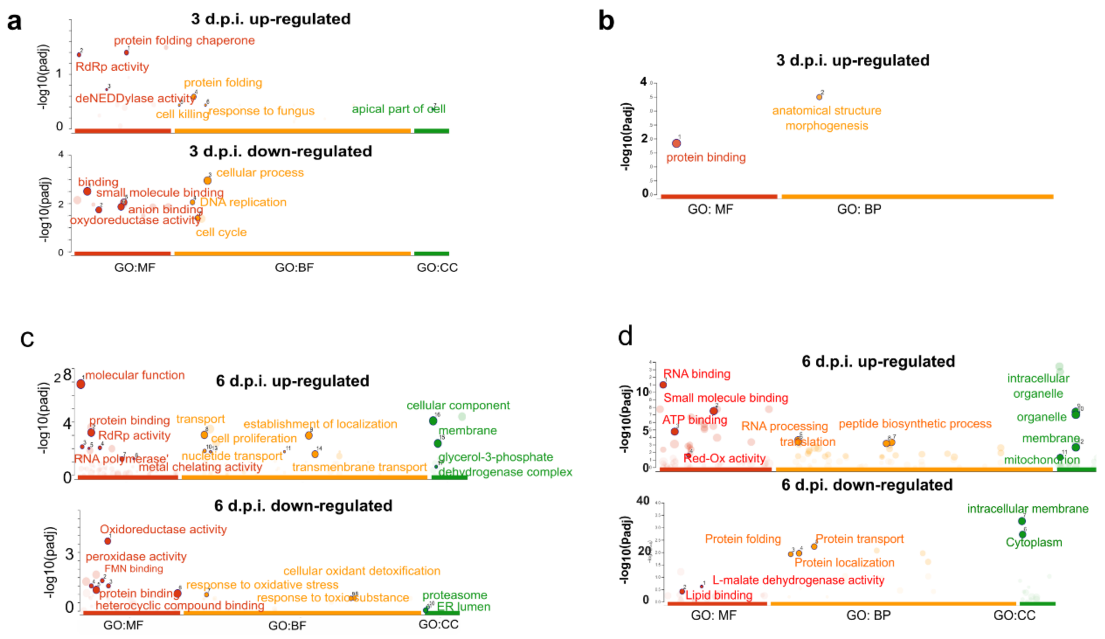

Supplementary Figure 3. Gene Ontology analysis of SFTSV infection in BME6 cells. a-b. Functional enrichment analysis of transcriptomic (a) and proteomic (b) at 3-day post-infection data using g:Profiler with default parameters (FDR = 0.05, user threshold p = 0.05). Categories of GO:Molecular Function (GO:MF coloured in red), GO:Biological Function (BF coloured in yellow), and GO:Cellular Compartment (CC coloured in green). The y-axis shows adjusted p-values. **c-d.** Functional enrichment analysis of transcriptomic (a) and proteomic (b) at 6-day post-infection data using g:Profiler with default parameters (FDR = 0.05, user threshold p = 0.05). Categories of GO:Molecular Function (GO:MF coloured in red), GO:Biological Function (BF coloured in yellow), and GO:Cellular Compartment (CC coloured in green). The y-axis shows adjusted p-values.

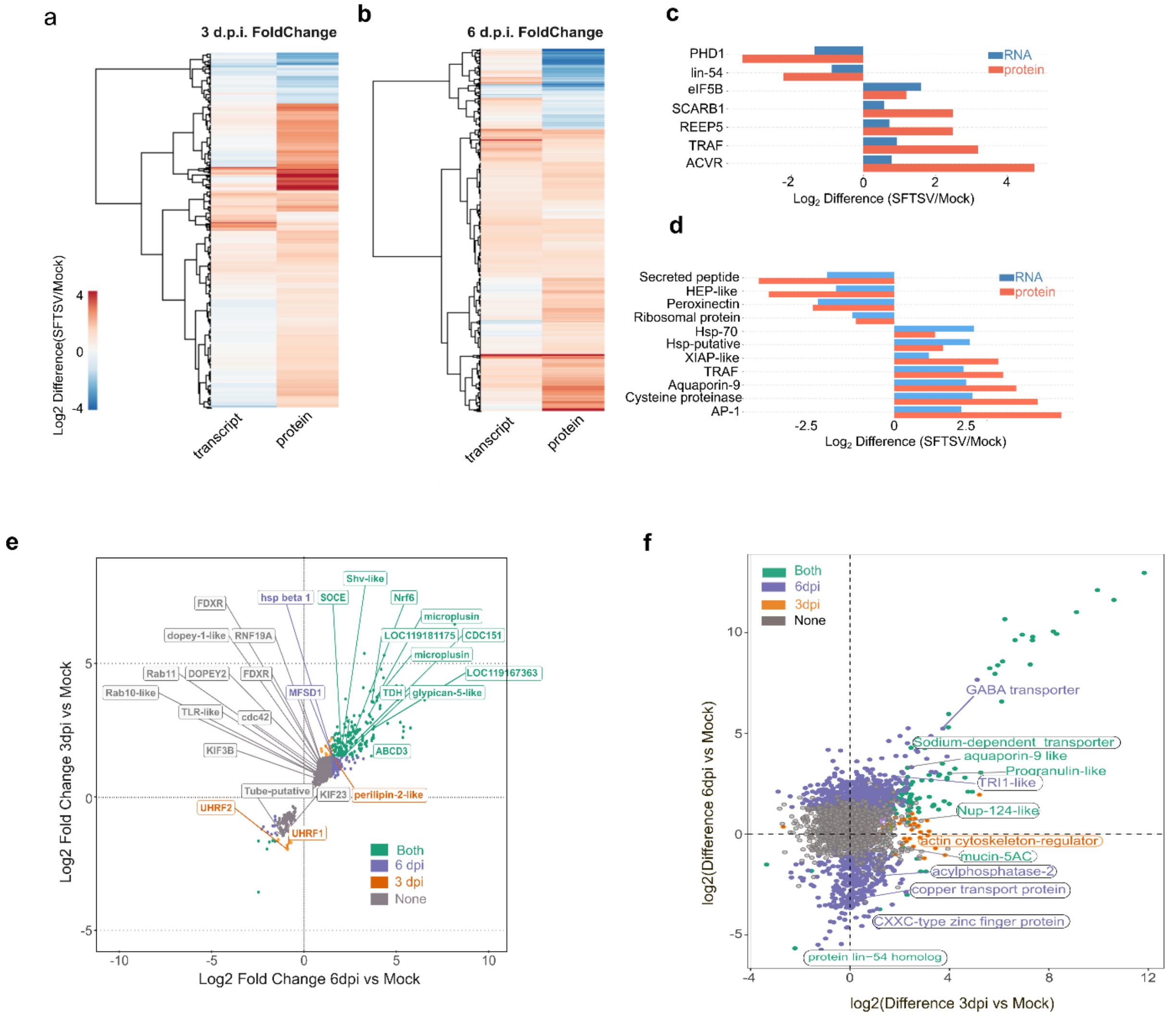

Supplementary Figure 4. Dynamics of SFTSV infection in BME6 infected cells using transcriptomics and proteomics. a–b. Hierarchical clustering of transcripts and proteins differentially expressed in BME/CTVM6 cells infected with SFTSV. Data are shown for 3 days post-infection (d.p.i.) **(a)** and 6 d.p.i. (**b**). Red indicates upregulated genes, blue indicates downregulated genes, and white indicates no significant change. **c–d.** Subset of genes showing concordant expression trends at both transcript and protein levels. Selected genes are shown for 3 d.p.i. (c) and 6 d.p.i. (d), with transcript regulation in red and protein regulation in blue. **e.** Scatter plot of transcripts with adjusted p-value (padj) ≤ 0.05. The x-axis shows $\log_2$ fold change (SFTSV 6 d.p.i. / mock), and the y-axis shows $\log_2$ fold change (SFTSV 3 d.p.i. / mock). Transcripts with significant changes only at 3 d.p.i. are shown in orange, only at 6 d.p.i. in purple, at both time points in green, and at neither in grey. **f.** Similar scatter plot for proteins with padj ≤ 0.05. The x-axis shows $\log_2$ difference (SFTSV 3 d.p.i. / mock) and the y-axis shows $\log_2$ difference (SFTSV 6 d.p.i. / mock). Proteins with significant changes only at 3 d.p.i. are shown in orange, only at 6 d.p.i. in purple, at both time points in green, and at neither in grey. Source data are provided in the accompanying source data file.

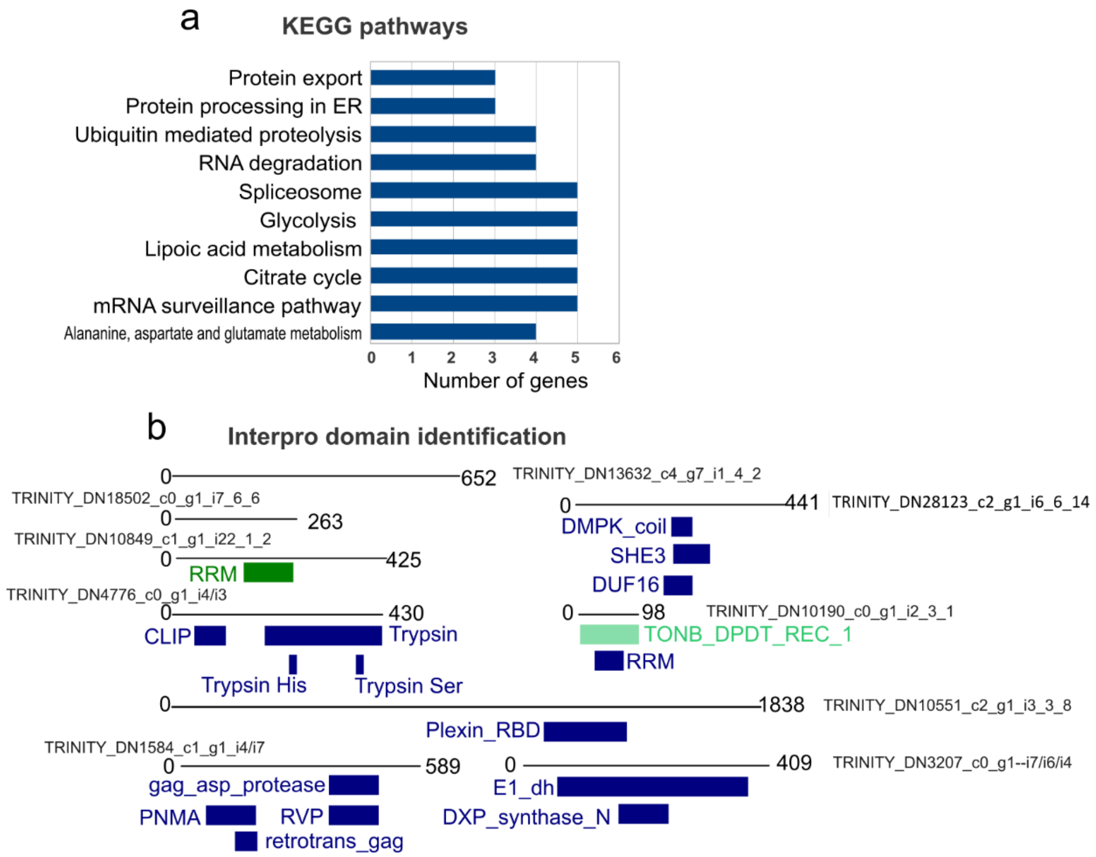

Supplementary Figure 5. SFTSV-N interactomics pathway enrichment and Protein domain characterization of uncharacterized interactors. **a**. KEGG pathway analysis combining all interactors of SFTSV-N proteins. **b.** Uncharacterized protein sequences were searched for PFAM protein domains (MOTIF Search shown in blue, and PROSITE shown in green). Identified domains with $p<0.05$ are represented (Sup. Table 5). RRM= RNA recognition motif; DMPK_coil= myotonic dystrophy protein kinase coil; SHE3= SWI5-dependent HO expression protein 3; DUF16= domain of unknown function; CLIP= clip domain; TONB_DPDT_REC_1= TonB-dependent receptors 1; Plexin_RBD=Plexin RhoGTPase-binding domain; PNMA= paraneoplastic antigens Ma-like; RVP= retroviral protease-like; E1_dh= dehydrogenase E1 component; DXP_synthase_N= 1-deoxy-D-xylulose-5-phosphate synthase. Source data are provided as source data file.

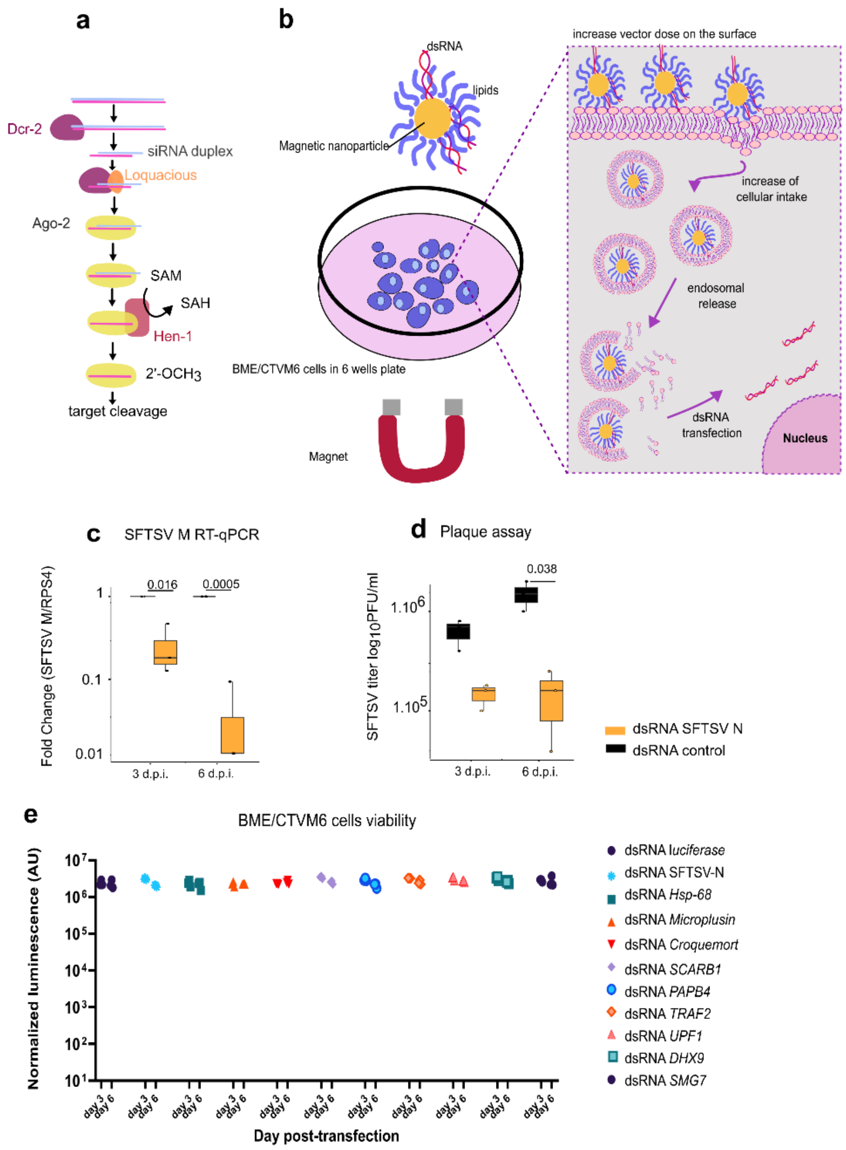

Supplementary Figure 6. dsRNA-induced silencing in BME6 cells. a. BME/CTVM6 siRNA pathway schematic. **b**. Schematic of the Magnetofection principles (Magnetofectamine^{TM} O2 kit, Oz Biosciences). **c–d**. BME/CTVM6 cells were transfected with SFTSV N-targeting dsRNA or control dsRNA (targeting *Renilla luciferase*) using Magnetofectamine™ O2 for 18 hours prior to infection with SFTSV at an MOI of 0.5 PFU/cell. **c.** Quantification of SFTSV segment M by RT-qPCR. Black boxes and dots represent control cells transfected with luciferase-targeting dsRNA, while yellow boxes and dots represent cells transfected with dsRNA targeting the SFTSV N gene. Fold change was normalized to the housekeeping gene *RPS4*. **d**. Quantification of infectious SFTSV particles by plaque assay at 3 and 6 days post-infection (d.p.i.). For panels **c–d**, center lines indicate medians; box limits represent the 25th and 75th percentiles; whiskers extend to 1.5 times the interquartile range. Boxplot statistics were generated using R software. Statistical significance was assessed using paired two-tailed Student's *t*-tests; significant p-values are indicated where applicable. **e**. Quantification of ATP present in dsRNA treated cells at 3- and 6-day post-transfection. All targets were transfected using Magnetofectamine^{TM} 02 and ATP was measure by Luminescence using Cell Glo Titer Promega kit, as measurement of BME/CTVM6 cells viability. All dsRNA from Figure 5, have been tested here, including dsRNA luciferase (control in dark purple circle), dsRNA-SFTSV-N (cyan star); dsRNA *Hsp-68* (blue square); dsRNA *Microplusin* (orange triangle); dsRNA *Croquemort* (red triangle); dsRNA *SCARB1* (light purple diamond); dsRNA *PAPB4* (blue circle); dsRNA *TRAF2* (orange diamond); dsRNA *UPF1* (pink triangle); dsRNA *DHX9* (green square); dsRNA *SMG7* (black circle). Statistical comparisons between dsRNA-luciferase-treated cells and other dsRNA conditions were performed using two-way ANOVA. Two-way ANOVA was also used to compare luminescence levels between 3 and 6 days post-infection.Source data are provided as source data file.

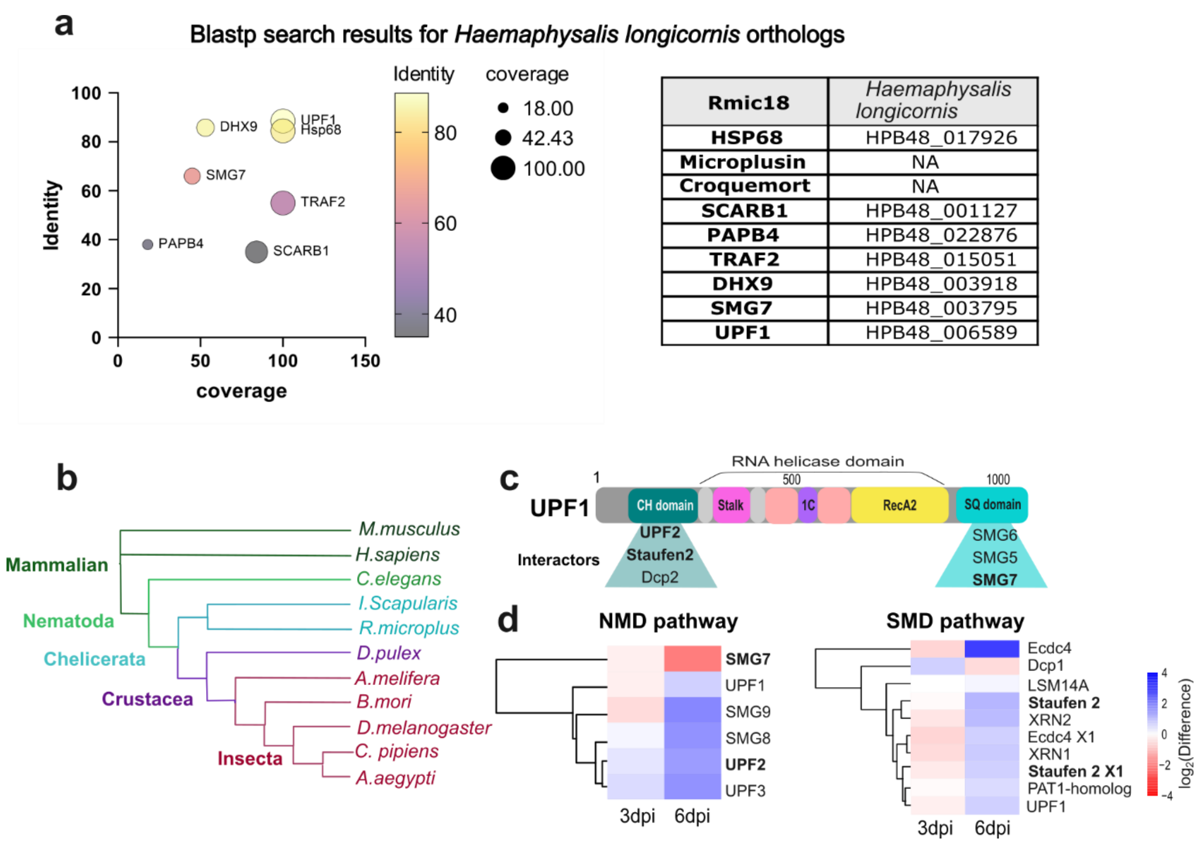

Supplementary Figure7. Importance of identified antiviral effectors for tick and SFTSV biology. a. BLASTp (protein–protein) search results for all RMIC18 targets tested in Figure 5, using *Haemaphysalis longicornis* (the main vector of SFTSV) as the reference database. Left panel: Bubble plot showing sequence coverage (circle size; larger indicates higher coverage) and identity (color-coded; yellow indicates high identity, grey indicates low identity) between RMIC18 proteins and their *Hae. longicornis* orthologs. Right panel: Table listing the corresponding *Hae. longicornis* protein identifiers for each ortholog. **b**. Phylogenetic tree of the UPF1 proteins in Mammalian, Nematodes, Chelicerates, Crustacea, and Insecta. **c.** Schematic representations of human UPF1, and mention of identified interactors with CH domain in dark green UPF2, Staufen, Dcp2, and in Cyan interactors of the SQ domain. **d.** Differential protein expression of NMD or SMD related protein found in the *R. microplus* proteome. Blue represents up-regulated protein expression and red represents down-regulated protein expression. Source data are provided as source data file.

Top hits	Blast results description (*R.microplus*)	Gene Symbol	Blast results ID (*R. microplus*)	Gene ref. (*R. microplus*)	Ortholog *I. Scapularis* (>60% identity)	*I. Scapularis* Uniprot ID	co-IP with SFTSV N at 3 d.p.i.	co-IP with SFTS V N at 6 d.p.i.
TRINITY_DN15242_c1_g1_i14_2_3	60S ribosomal protein L10A	60SR_L10A	XP_037272253.1	LOC119164217	ISCW015602	B7P3X5	+	+
TRINITY_DN96811_c0_g2_i3_6_16;TRINITY_DN13497_c1_g1_i8_2_11	60S ribosomal protein L7-like isoform X1	60SR_L7	XP_037277595.1	LOC119170505			+	+
TRINITY_DN62760_c0_g1_i3_5_21;TRINITY_DN44310_c0_g1_i11_3_1;TRINITY_DN44310_c0_g1_i13_1_2;TRINITY_DN44310_c0_g1_i8_2_2;TRINITY_DN62760_c0_g1_i10_5_21	(Lyso)-N-acylphosphatidylethanolamine lipase-like	ABDH4	XP_037284146.1	LOC119176910	ISCW008036	B7PSU1	+	
TRINITY_DN3214_c0_g1_i3_4_1	ATP-dependent RNA helicase A-like isoform X1	DHX9	XP_037270024.1	LOC119161586	ISCW009103	B7PZF0	+	+
TRINITY_DN3362_c0_g1_i7_3_3;TRINITY_DN3362_c0_g1_i5_3_3	DCC-interacting protein 13-alpha-like isoform	DIP13A	XP_037280973.1	LOC119174246	ISCW020843	B7PX84	+	+
TRINITY_DN5624_c0_g1_i19_2_6;TRINITY_DN5624_c0_g1_i6_2_6	general transcription and DNA repair factor IIH helicase subunit	ERCC3	XP_037285925.1	LOC119178783	ISCW004030		+	
TRINITY_DN4781_c1_g1_i74_6_7;TRINITY_DN4781_c1_g1_i7_6_10;TRINITY_DN4781_c1_g1_i55_6_14	exocyst complex component 2-like isoform	EXOC2	XP_037283724.1	LOC119176507	ISCW006643	B7PR09	+	+
TRINITY_DN2978_c3_g1_i3_5_11	GMP reductase 2-like	GMPR2	XP_037284350.1	LOC119177074	ISCW019974	B7PTS0	+	+
TRINITY_DN7877_c0_g1_i14_6_2	insulin-like growth factor 2 mRNA-binding protein 1	IGF2BP1	XP_037285919.1	LOC119178779	ISCW009590	B7Q3I5	+	
TRINITY_DN76530_c0_g2_i19_1_22;TRINITY_DN76530_c0_g2_i3_3_3;TRINITY_DN76530_c0_g1_i5_5_6;TRINITY_DN76530_c0_g1_i19_5_6;TRINITY_DN76530_c0_g1_i13_5_9	inositol polyphosphate 5-phosphatase K-like isoform	INPP5K	XP_037282024.1	LOC119174953	ISCW016108	B7P165	+	+
TRINITY_DN23339_c0_g2_i2_4_7	neurotactin-like	Neurotactin	XP_037277880.1	LOC119170734	ISCW013301	B7QA70	+	
TRINITY_DN11180_c1_g1_i7_3_11;TRINITY_DN11180_c1_g1_i5_3_11;TRINITY_DN11180_c1_g1_i4_3_11;TRINITY_DN11180_c1_g1_i3_3_11;TRINITY_DN11180_c0_g1_i6_4_11;TRINITY_DN11180_c0_g1_i5_4_11;TRINITY_DN11180_c0_g1_i1_6_11	NF-kappa-B-repressing factor isoform	NKRF	XP_049517890.1				+	
TRINITY_DN17563_c0_g1_i3_6_14;TRINITY_DN48125_c0_g1_i6_2_4;TRINITY_DN17563_c0_g1_i2_6_5	succinyl-CoA:3-ketoacid coenzyme A transferase 1, mitochondrial-like	OXCT	XP_037277435.1	LOC119170392	ISCW002707	B7P8K3	+	+
TRINITY_DN23479_c0_g1_i11_3_26	polyadenylate-binding protein 4-like	PABP-4	XP_037274389.1	LOC119167073	ISCW014211	B7QI53	+	
TRINITY_DN11687_c3_g3_i5_5_1	parafibromin	Parafibromin	XP_037269097.1	LOC119160921	ISCW008023	B7PWD3	+	+
TRINITY_DN8340_c1_g1_i7_1_1	zinc finger protein on ecdysone puffs-like	PEP	XP_037269253.1	LOC119161035			+	
TRINITY_DN28124_c0_g1_i5_4_1;TRINITY_DN56804_c0_g1_i20_3_20;TRINITY_DN56804_c0_g1_i1_1_6;TRINITY_DN28124_c0_g1_i15_4_1;TRINITY_DN56804_c0_g1_i18_2_1	transcriptional activator protein Pur-beta-like isoform X1	PURB	XP_037287686.1	LOC119180669	ISCW005651	B7PKC2	+	
TRINITY_DN3723_c0_g1_i3_6_1	PX domain-containing protein kinase-like protein isoform X1	PXK	XP_037282205.1				+	+
TRINITY_DN31792_c0_g2_i60_6_6;TRINITY_DN31792_c0_g2_i39_4_4;TRINITY_DN31792_c0_g2_i11_6_4;TRINITY_DN31792_c0_g1_i1_3_5	splicing factor 3B subunit 3-like	SF3B3	XP_037283304.1	LOC119176231	ISCW020447	B7PXI3	+	
TRINITY_DN17409_c69_g1_i1_6_3;TRINITY_DN2327_c0_g2_i1_1_1	N protein SFTSV	SFTSV_N	XP_037269888.1				+	+
TRINITY_DN23455_c1_g2_i1_2_10;TRINITY_DN23455_c1_g2_i5_2_9;TRINITY_DN1947_c0_g1_i14_5_34	Helicase SKI2W	SKI2W	XP_037269888.1	LOC119161489	ISCW013335	B7QFE6	+	+
TRINITY_DN50074_c0_g4_i2_5_2;TRINITY_DN50074_c0_g4_i1_5_2	small nuclear ribonucleoprotein G	snRNP	XP_037279177.1	LOC119172244	ISCW008104	B7PTI1	+	+
TRINITY_DN644_c0_g1_i9_4_8;TRINITY_DN644_c0_g1_i5_6_8	signal recognition particle 14 kDa protein-like	SRP_14kDA	XP_037274344.1	LOC119167038	ISCW012442	B7QAY0	+	+
TRINITY_DN41237_c0_g1_i43_6_5;TRINITY_DN41237_c0_g1_i16_6_5;TRINITY_DN41237_c0_g1_i18_6_8	pre-mRNA-splicing factor syf1 homolog	Syf1	XP_037291691.1	LOC119187711	ISCW023297	B7QIT4	+	+
TRINITY_DN25190_c0_g1_i14_1_5	phosphatidate cytidylyltransferase, mitochondrial	TAMM41	XP_037498275.1				+	
TRINITY_DN33447_c3_g1_i8_5_4;TRINITY_DN73448_c0_g1_i8_2_3;TRINITY_DN73448_c0_g1_i22_1_6;TRINITY_DN73448_c0_g1_i43_1_7;TRINITY_DN73448_c0_g1_i30_2_4;TRINITY_DN73448_c0_g1_i39_2_4;TRINITY_DN33447_c3_g1_i5_6_2	TATA element modulatory factor-like	TMF1	XP_037287410.1	LOC119180331			+	
TRINITY_DN7022_c0_g1_i3_4_2;TRINITY_DN7022_c0_g1_i2_4_2;TRINITY_DN4503_c0_g1_i4_2_2;TRINITY_DN4503_c0_g1_i3_2_2;TRINITY_DN4503_c0_g1_i5_3_1	TNF receptor-associated factor 2-like	TRAF2	XP_037271015.1	LOC119163172	ISCW014589	B7QJX9	+	+
TRINITY_DN16764_c0_g1_i1_6_5;TRINITY_DN1487_c0_g1_i9_2_28	ubiquitin-conjugating enzyme E2 G2	UbE2G2	XP_037282056.1	LOC119174998	ISCW019512	B7PT81	+	
TRINITY_DN8723_c0_g1_i2_5_19;TRINITY_DN8723_c0_g1_i3_5_3;TRINITY_DN19240_c0_g1_i1_3_3	regulator of nonsense transcripts 1	UPF1	XP_037289429.1	LOC119182959	ISCW010285	B7PZ74	+	+
TRINITY_DN116646_c0_g2_i2_3_4;TRINITY_DN116646_c0_g2_i1_2_2;TRINITY_DN116646_c0_g2_i9_2_2;TRINITY_DN116646_c0_g2_i3_2_6;TRINITY_DN116646_c0_g2_i14_1_4;TRINITY_DN116646_c0_g1_i12_6_3	Valacyclovir hydrolase	VCH	XP_037283312.1	LOC119176235			+	
TRINITY_DN442282_c0_g1_i5_1_1;TRINITY_DN442282_c0_g3_i1_4_4	hypothetical protein HPB51_003695		KAH8032911.1				+	

tr|F1BA48|F1BA48_SFTSV;tr|A0A0B5A9D7|A0A0B5A9D7_SFTSV;sp|P0DW82|NCAP_SFTSV	N cap SFTSV						+	+
TRINITY_DN433978_c1_g1_i1_6_1	nucleocapside Protein						+	+
TRINITY_DN9626_c0_g1_i5_2_20;TRINITY_DN12_c156_g1_i9_5_26;TRINITY_DN12_c156_g1_i7_4_26;TRINITY_DN12_c156_g1_i4_5_53;TRINITY_DN12_c156_g1_i1_5_53	ORF4 IRE19 Rhabdo						+	+
TRINITY_DN399864_c0_g1_i1_6_1	putative N protein Rhabdo IRE19						+	+
tr|F1BA46|F1BA46_SFTSV;tr|A0A0B5AEH2|A0A0B5AEH2_SFTSV;TRINITY_DN33733_c1_g1_i2_1_1;TRINITY_DN60572_c130_g1_i1_5_5	RdRp SFTSV						+	+
TRINITY_DN28123_c2_g1_i6_6_14	Uncharacterized						+	+
TRINITY_DN10190_c0_g1_i2_3_1	Uncharacterized						+	+
TRINITY_DN10551_c2_g1_i3_3_8	Uncharacterized						+	+
TRINITY_DN3207_c0_g1_i7_5_63;TRINITY_DN3207_c0_g1_i6_5_42;TRINITY_DN3207_c0_g1_i4_4_24;TRINITY_DN3207_c0_g1_i12_5_44	Uncharacterized						+	+
TRINITY_DN1584_c1_g1_i4_4_5;TRINITY_DN1584_c1_g1_i7_4_11;TRINITY_DN1584_c1_g1_i5_4_6;TRINITY_DN28413_c0_g1_i7_3_4;TRINITY_DN28413_c0_g1_i15_3_17;TRINITY_DN28413_c0_g1_i18_2_8	Uncharacterized						+	
TRINITY_DN4776_c0_g1_i4_5_52;TRINITY_DN4776_c0_g1_i3_4_10;TRINITY_DN25524_c0_g1_i2_1_7;TRINITY_DN25524_c0_g1_i5_1_7	Uncharacterized						+	
TRINITY_DN6317_c0_g1_i9_4_5;TRINITY_DN3995_c0_g1_i3_1_3;TRINITY_DN3995_c0_g1_i12_2_3	ATP-binding cassette sub-family D member 1-like	ABCD1	XP_037271484.1	LOC119163566	ISCW018310	B7PGT9		+
TRINITY_DN2027_c1_g1_i1_6_4;TRINITY_DN2027_c0_g1_i7_1_12;TRINITY_DN2027_c0_g1_i3_2_3	B-cell receptor-associated protein 31-like isoform X2	BCAP31	XP_037281361.1	LOC119174524	ISCW009367	B7PYE7		+
TRINITY_DN5687_c3_g2_i1_5_5	blood vessel epicardial substance-like	BVES	XP_037281702.1	LOC119174760				+
TRINITY_DN13488_c0_g1_i68_6_20;TRINITY_DN13488_c0_g1_i65_6_24;TRINITY_DN13488_c0_g1_i55_6_18;TRINITY_DN13488_c0_g1_i36_6_24;TRINITY_DN13488_c0_g1_i17_6_15	Golgi complex subunit 5	COG5	XP_037498936.1					+
TRINITY_DN20188_c0_g1_i2_3_1;TRINITY_DN20188_c0_g1_i10_3_1;TRINITY_DN49084_c0_g1_i5_5_1;TRINITY_DN20188_c0_g1_i11_3_1	cleavage and polyadenylation specificity factor subunit 6-like isoform X4	CPSF6	XP_037277672.1					+
TRINITY_DN23044_c0_g1_i9_2_1;TRINITY_DN23044_c0_g1_i6_1_44;TRINITY_DN23044_c0_g1_i5_1_59;TRINITY_DN23044_c0_g1_i3_1_70;TRINITY_DN23044_c0_g1_i1_1_8;TRINITY_DN10638_c0_g1_i43_6_30;TRINITY_DN10638_c0_g1_i37_6_76;TRINITY_DN10638_c0_g1_i33_6_44;TRINITY_DN10638_c0_g1_i3_6_63;TRINITY_DN10638_c0_g1_i29_6_31	elongation factor Tu-like	EF-tu	XP_037268029.1	LOC119159389	ISCW000086	B7P0L0		+
TRINITY_DN2685_c1_g2_i1_6_25	ESF1 homolog	ESF1	XP_037287280.1	LOC119180240	ISCW023079	B7QLZ1		+
TRINITY_DN4705_c0_g1_i3_2_8;TRINITY_DN4705_c0_g1_i2_2_8;TRINITY_DN4400_c0_g1_i3_5_9;TRINITY_DN4400_c0_g1_i1_5_9	four and a half LIM domains protein 2-like isoform X4	FHL2	XP_037280949.1	LOC119174226	ISCW016762	B7PDH9		+
TRINITY_DN3367_c0_g1_i27_1_9;TRINITY_DN3367_c0_g1_i21_1_47;TRINITY_DN3367_c0_g1_i12_2_67;TRINITY_DN3367_c0_g1_i23_2_67;TRINITY_DN743_c7_g1_i40_5_58;TRINITY_DN743_c7_g1_i7_5_58;TRINITY_DN3367_c0_g1_i4_2_67;TRINITY_DN743_c7_g1_i21_5_58;TRINITY_DN743_c7_g1_i12_5_3;TRINITY_DN743_c7_g1_i30_6_58;TRINITY_DN743_c7_g1_i6_6_2;TRINITY_DN743_c7_g1_i27_6_1	germinal-center associated nuclear protein-like	GANP	XP_037280306.1	LOC119173606	ISCW022606	B7QDA7		+
TRINITY_DN40849_c0_g1_i6_2_1;TRINITY_DN45727_c0_g1_i1_4_5;TRINITY_DN40849_c0_g1_i3_1_9	glutamine synthetase-like	GS	XP_037291891.1	LOC119187887	ISCW018771	B7PNU5		+
TRINITY_DN15061_c0_g1_i2_1_59	m7GpppX diphosphatase-like	m7GpppX diphosphatase	XP_037271565.1	LOC119163629	ISCW010384	B7Q4B4		+
TRINITY_DN1158_c0_g1_i8_5_2	iron-sulfur protein NUBPL-like	NUBPL	XP_037269941.1	LOC119161522	ISCW009337	B7Q0G0		+
TRINITY_DN977_c1_g1_i4_6_79;TRINITY_DN977_c1_g1_i27_6_74;TRINITY_DN977_c1_g1_i22_6_144;TRINITY_DN19220_c0_g1_i1_3_12;TRINITY_DN19220_c0_g1_i2_3_27;TRINITY_DN19220_c0_g1_i6_2_21;TRINITY_DN19220_c0_g1_i4_3_15	uridine diphosphate glucose pyrophosphatase NUDT14	NUDT14	XP_037500874.1					+
TRINITY_DN62_c5_g1_i6_3_4;TRINITY_DN62_c5_g1_i12_3_4;TRINITY_DN62_c5_g1_i7_3_4;TRINITY_DN62_c5_g1_i18_3_4	nuclear pore complex protein Nup214-like isoform X3	NUP124	XP_037269628.1	LOC119161321	ISCW000204	B7P6T1		+
TRINITY_DN4456_c1_g1_i5_1_1	2-oxoglutarate dehydrogenase, mitochondrial-like	OGDHL	XP_037269250.1	LOC119161032	ISCW003165	B7PCU5		+
TRINITY_DN8689_c2_g1_i3_3_9;TRINITY_DN8689_c2_g1_i2_3_9;TRINITY_DN8689_c2_g1_i1_3_9;TRINITY_DN18750_c0_g3_i2_6_6	osteoclast-stimulating factor 1-like	OSTF1	XP_037291117.1					+
TRINITY_DN15069_c0_g2_i10_5_20;TRINITY_DN15069_c0_g2_i11_5_8;TRINITY_DN15069_c0_g1_i4_2_9	piezo-type mechanosensitive ion channel component 1-like isoform X1	PIEZO1	XP_037284738.1	LOC119177358	ISCW010459	B7Q5S1		+
TRINITY_DN5278_c8_g1_i5_5_1;TRINITY_DN5278_c8_g1_i7_4_1;TRINITY_DN6300_c0_g1_i6_3_11	piwi-like protein 1	PIWI1L	XP_037269397.1	LOC119161148	ISCW012408	B7QEM1		+
TRINITY_DN4656_c0_g1_i1_1_4	protein arginine N-methyltransferase 6-	PRMT6	XP_037281958.1	LOC119174927	ISCW000176	B7P3U8		+

	like							
TRINITY_DN5721_c0_g2_i1_6_3	proteasome activator complex subunit 4-like	PSME4	XP_037274192.1	LOC119166906	ISCW020904	B7Q408		+
TRINITY_DN14447_c0_g1_i1_1_3	retinal dehydrogenase 2-like	RALDH2	XP_037292015.1	LOC119188190	ISCW012082	B7QAL5		+
TRINITY_DN16107_c0_g1_i9_3_27;TRINITY_DN16107_c0_g1_i31_2_25;TRINITY_DN16107_c0_g1_i24_2_29	ralBP1-associated Eps domain-containing protein 1-like isoform X3	REPS1	XP_037281094.1	LOC119174336	ISCW016119	B7P2S6		+
TRINITY_DN5032_c0_g1_i7_4_3;TRINITY_DN5032_c0_g1_i3_6_3	reticulon-4-interacting protein 1, mitochondrial-like	RTN4IP1	XP_037281967.1	LOC119174935	ISCW002789	B7PCR9		+
TRINITY_DN5000_c0_g1_i11_4_5;TRINITY_DN17047_c0_g1_i8_2_10;TRINITY_DN5000_c0_g1_i5_4_9;TRINITY_DN17047_c0_g1_i6_2_9	succinate dehydrogenase assembly factor 2-A, mitochondrial-like isoform X1	SDH2-A	XP_037276068.1	LOC119169087	ISCW001947	B7P7L1		+
TRINITY_DN675_c2_g1_i6_1_4;TRINITY_DN675_c2_g1_i10_1_4	protein SMG7-like isoform X1	SMG7	XP_037269345.1	LOC119161110	ISCW003762			+
TRINITY_DN39884_c0_g1_i6_1_8;TRINITY_DN39884_c0_g1_i22_2_10	signal recognition particle 14 kDa protein-like	SRP_14kDA	XP_037274344.1	LOC119167038	ISCW012442	B7QAY0		+
TRINITY_DN9978_c0_g1_i8_2_5;TRINITY_DN9978_c0_g1_i9_3_4	signal recognition particle 9 kDa protein-like	SRP_9Kda	XP_037274853.1	LOC119167475	ISCW008988	B7Q3G1		+
TRINITY_DN3587_c0_g1_i8_1_6;TRINITY_DN3587_c0_g1_i6_1_6;TRINITY_DN1792_c2_g1_i6_6_5	3'-5' ssDNA/RNA exonuclease TatD-like	tAtD-like	XP_037279490.1					+
TRINITY_DN17589_c0_g1_i2_1_1;TRINITY_DN389_c4_g1_i6_5_1	transcription factor A, mitochondrial-like isoform X1	TFAM	XP_037291770.1	LOC119187790				+
TRINITY_DN11287_c0_g1_i4_3_7;TRINITY_DN11287_c0_g1_i1_3_7	T-cell immunomodulatory protein-like partial	Tip	XP_037271101.1	LOC119163247	ISCW006297	B7PQP3		+
TRINITY_DN275_c1_g2_i10_1_1;TRINITY_DN6303_c0_g1_i11_4_8;TRINITY_DN6303_c0_g1_i25_5_8	tubulin--tyrosine ligase-like protein 12 isoform X2	TTLL12	XP_037274013.1	LOC119166777	ISCW008116			+
TRINITY_DN9074_c0_g1_i9_4_1	tubulin beta chain-like	TUBB	XP_037272508.1	LOC119164422	ISCW017133	B7PA92		+
TRINITY_DN101785_c0_g2_i1_4_6;TRINITY_DN101785_c0_g1_i1_1_6	vacuolar protein sorting-associated protein 8 homolog	Vps8	XP_037279427.1	LOC119172428	ISCW010903	B7Q5P5		+
TRINITY_DN399864_c0_g1_i5_6_1	N protein IRE19 virus							+
TRINITY_DN8916_c0_g2_i1_6_42;TRINITY_DN12431_c0_g1_i55_1_24;TRINITY_DN12431_c0_g1_i4_2_29;TRINITY_DN12431_c0_g1_i29_1_23;TRINITY_DN12431_c0_g1_i2_3_18;TRINITY_DN12431_c0_g1_i11_3_18	N protein IRE19 virus							+
TRINITY_DN9626_c0_g1_i5_1_3;TRINITY_DN12_c156_g1_i9_6_2;TRINITY_DN12_c156_g1_i7_5_2;TRINITY_DN12_c156_g1_i4_6_23;TRINITY_DN12_c156_g1_i1_6_24;TRINITY_DN401624_c4_g1_i1_1_1;TRINITY_DN513896_c0_g1_i1_6_1;TRINITY_DN411874_c1_g1_i1_5_2;TRINITY_DN73363_c5_g1_i1_6_1	N protein IRE19 virus							+
TRINITY_DN9626_c244_g1_i1_1_1;TRINITY_DN31471_c8_g1_i1_3_1	N protein IRE19 virus							+
TRINITY_DN444306_c2_g1_i1_2_1	Non-structural protein SFTSV							+
TRINITY_DN356050_c4_g1_i1_1_1	nucleocapside Protein							+
TRINITY_DN5618_c31_g1_i1_2_1	ORF4 IRE19 Rhabdo							+
TRINITY_DN10849_c1_g1_i22_1_2	Uncharacterized							+
TRINITY_DN18502_c0_g1_i7_6_6;TRINITY_DN18502_c0_g1_i4_5_6;TRINITY_DN18502_c0_g1_i1_4_9;TRINITY_DN12773_c0_g1_i3_2_10;TRINITY_DN12773_c0_g1_i2_2_10	Uncharacterized							+
TRINITY_DN13632_c4_g7_i1_4_2;TRINITY_DN13632_c4_g3_i1_2_2	Uncharacterized							+

Supplementary Table 1. Differential Scoring of SFTSV N-BME6 Cell Interactome. SFTSV N-BME6 cells protein-protein interactions were scored using Tubulin pull down as control condition. Significant interactors were determined by two-tailed t-tests with permutation-based false discovery rate statistics. We performed 250 permutations, and the FDR threshold was set at 0.05.

primer	ribosomal RNA target	primer sequence 5'-3'
probe 1	18S	AGGCAGACACTTGAAAGAAACGTCG
probe 2	18S	AAAGAACCGGCATGATGTTCAGTCC
probe 3	18S	TGTACAAAGGGCAGGGACGTAATCA
probe 4	18S	CGTCCGTCCCTCTTAATCATTAC
probe 5	18S	GATCCTTCCGCAGGTTCAC
probe 6	18S	ACATCTAAGGGCATCACAGAC
probe 7	18S	CGTCACTACCTCCCCG
probe 8	18S	GTATACGCTATTGGAGCTGG
probe 9	18S	CTCCACTCCTGGTGGTG
probe 10	18S	AATCTGTCAATCCTCCCAGTGTCCG
probe 11	IST1	CCTAACCAGAAAATGCCTGCGGTAC
probe 12	IST1	CTCGTTCTTCCGTGCTTCCTCTTTC
probe 13	IST1	GAAAGCCGACCTATGAGTGCGTTG
probe 14	IST1	GAGCAAACGGTGTGTTTC
probe 15	IST1	TACTTTAGCCAACAGGTGGAG
probe 16	IST1	CATCGCCGTGCCATG
probe 17	IST1	CAGCCTCGCCTTCTACC
probe 18	IST1	GCTCCACATTCAGGCAG
probe 19	IST1	GCTTGTGCCGTTCCTCTCGTACTTG
probe 20	IST1	CAATTCCGTACAGTCAAAACCAAACGT
probe 21	5.8S	CCTGCAATTCACACCAAGTC
probe 22	5.8S	CTCAGACAGACGAAGCCAAGGGAA
probe 23	IST2	CGAACGTTCCCTGTTCG
probe 24	IST2	GAGTACGCAACCTCGAC
probe 25	IST2	GTGTTTACCGTGCGTTAAAGTTGCG
probe 26	IST2	CGACGTCGCTATGAACGCTTGG
probe 27	IST2	GACCGTGTCGAAAGCCCAAACA
probe 28	28S	TTGTGAACTATCGGTCTCTCGGTCG
probe 29	28S	ATTGGTCTTTCGCCCCTATACCCG
probe 30	28S	TGTCTTAAGCAACCAACACCCTTCA
probe 31	28S	CTAAGGACCGACTGACCCATGTTCA
probe 32	28S	GAGAAAAGAAAACTCTTCCCGGGGC
probe 33	28S	GATCCGTTTTGCCGACTTCCCTTAC
probe 34	28S	CTTCTTTCCCCGCTGATTTTGCCAA
probe 35	28S	ATTGACTCGCGCACATGTTAGACTC
probe 36	28S	GTTACCGTTTGACAGATGTACCGCC
probe 37	28S	CGACGTCGCTATGAACGCTTGG
probe 38	28S	CGATGAGAGTAGTGGTATTTCACTTG
probe 39	28S	CCACAAGAGAGTCATAGTTACTCC
probe 40	28S	CGTGCCAGTTCTGAGTTG
probe 41	28S	GGATTTTCAAGGGCCGAC
probe 42	28S	CTCGTCGCGGCTTAG
probe 43	28S	CCGTACACCCATTGAAAGTTTG
probe 44	28S	GTTCACCATCTTTCGGGTG
probe 45	28S	GTTCTCCGCTCCGTTTC
probe 46	28S	CAGGTCACCTCCACTG

Supplementary Table 2. Ribosomal RNA depletion primers. List of primers targeting ribosomal RNA of *Rhipicephalus microplus*. Primers are targeting 18S, 28S, 5.8S and IST1, IST2 identified in RMIC18 genome.
