# Supplementary material for: Multi-omics analysis of SFTS virus infection in Rhipicephalus microplus cells reveals antiviral tick factors
Source: Nat Commun. 2025 May 21;16:4732. doi: 10.1038/s41467-025-59565-w (PMC12095547; doi:10.1038/s41467-025-59565-w)
Supplement: Supplementary file 2 — Description of Additional Supplementary Files [file 41467_2025_59565_MOESM2_ESM.docx]

Description of Additional Supplementary Files

**File Name:** Supplementary Data 1

**Description:** Fasta files containing the Trinity-predicted gene isoforms.

**File Name:** Supplementary Data 2

**Description:** Fasta files containing the predicted proteome for Rhipicephalus microplus BME6 cells used in this study.

**File Name:** Supplementary Data 3

**Description:** Differential protein expression data comparing SFTSV-infected BME6 cells at 3 and 6 d.p.i. to mock-infected cells, with integrated annotation results (BLAST, EggNOG, InterProScan). Significantly upregulated and downregulated proteins were identified using a modified t-test (Perseus "one-sample, two-tailed t-test" with s₀ = 0.02 and BenjaminiHochberg FDR ≤ 0.05).

**File Name:** Supplementary Data 4

**Description:** Differential Trinity ID genes of BME/CTVM6 cells infected with SFTSV MOI 1 PFU/ml. Table include annotation of BME/CTVM6 Trinity genes including results from BLAST searches (Trinotate), ortholog searches (EggNOG) and protein domain searches (InterProScan). Differential gene expression analysis was performed using DESeq2, which models count data from RNA-seq experiments using negative binomial generalized linear models. Genes with an adjusted p-value (Benjamini–Hochberg correction) below 0.05 were considered significantly differentially expressed.

**File Name:** Supplementary Data 5

**Description:** Sqlite database built using PASA-EVM workflow containing novel alternative splicing events identified in the BME6 transcriptome (Figshare DOI: 10.6084/m9.figshare.25637232).

**File Name:** Supplementary Data 6

**Description:** Protein domain identification for uncharacterized SFTSV-N interactors shown in Figure 4.
