# Supplementary material for: Multi-omics analysis of SFTS virus infection in Rhipicephalus microplus cells reveals antiviral tick factors
Source: Nat Commun. 2025 May 21;16:4732. doi: 10.1038/s41467-025-59565-w (PMC12095547; doi:10.1038/s41467-025-59565-w)
Supplement: Supplementary file 8 — Reporting Summary [file 41467_2025_59565_MOESM8_ESM.pdf]

nature portfolio

Corresponding author(s): Marine J. Petit; Benjamin Brennan; Alain Kohl

Last updated by author(s): Mar 11, 2025

Reporting Summary

Nature Portfolio wishes to improve the reproducibility of the work that we publish. This form provides structure for consistency and transparency in reporting. For further information on Nature Portfolio policies, see our Editorial Policies and the Editorial Policy Checklist.

Statistics

For all statistical analyses, confirm that the following items are present in the figure legend, table legend, main text, or Methods section.

n/a	Confirmed	
☐	☒	The exact sample size (*n*) for each experimental group/condition, given as a discrete number and unit of measurement
☐	☒	A statement on whether measurements were taken from distinct samples or whether the same sample was measured repeatedly
☐	☒	The statistical test(s) used AND whether they are one- or two-sided<br>*Only common tests should be described solely by name; describe more complex techniques in the Methods section.*
☒	☐	A description of all covariates tested
☐	☒	A description of any assumptions or corrections, such as tests of normality and adjustment for multiple comparisons
☐	☒	A full description of the statistical parameters including central tendency (e.g. means) or other basic estimates (e.g. regression coefficient) AND variation (e.g. standard deviation) or associated estimates of uncertainty (e.g. confidence intervals)
☐	☒	For null hypothesis testing, the test statistic (e.g. *F*, *t*, *r*) with confidence intervals, effect sizes, degrees of freedom and *P* value noted<br>*Give P values as exact values whenever suitable.*
☒	☐	For Bayesian analysis, information on the choice of priors and Markov chain Monte Carlo settings
☒	☐	For hierarchical and complex designs, identification of the appropriate level for tests and full reporting of outcomes
☐	☒	Estimates of effect sizes (e.g. Cohen's *d*, Pearson's *r*), indicating how they were calculated

Our web collection on statistics for biologists contains articles on many of the points above.

Software and code

Policy information about availability of computer code

Data collection	Illumina NovaSeq was used for RNA sequencing.<br>nanoElute (plugin v1.1.0.27, Brucker) coupled with a timsTOF Pro was used for interactomic data acquisition.<br>Orbitrap Exploris 480 was used for DIA proteomic data acquisition.<br>Image acquisition was performed on Zeiss LSM 710 Meta confocal microscope.<br>Promega CellTiter-Glo luminescence was acquired on CLARIOstar Plus plate reader (BMG Labtech)<br>Gene expression, measured by qPCR was collected on QuantStudio 3 RT-PCR system (ThermoFisher Scientific).
Data analysis	For RNA sequencing analysis we used the following softwares:<br>FastQC (v0.11.5)<br>Trinity (v2.14.0)<br>Transdecoder (v5.7.0)<br>STAR (v2.5.2b)<br>HTseq (v0.6.1)<br>DESeq2 Bioconductor package (v1.30.1)<br><br>Softwares used for proteomics analyses:<br>Perseus (v1.6.15.0)<br>Spectronaut (v16.2.220903.5300)<br>Proteowizard

MaxQuant (v1.6.17.0)
Cytoscape (v3.10.0)

Proteomics informed by Transcriptomics (PIT) and genome annotation softwares:
Diamond (v2.1.9)
eggNOG (v4.5)
InterProScan 5
Program to assemble spliced alignments PASA (v2.5.2)
EVidence Modeler
Augustus (v3.5.0)
SNAP (https://github.com/KorfLab/SNAP)
exonerate (v2.4.0)
miniprot (v0.12)
genome threader (v1.7.3)
taxonomy pipeline (https://github.com/stenglein-lab/taxonomy_pipeline/)
g:Profiler

Statistical analysis softwares and data visualization:
MEGA 6 Software
GraphPad Prism 10
R (v.4.3.0)
ImageJ

For manuscripts utilizing custom algorithms or software that are central to the research but not yet described in published literature, software must be made available to editors and reviewers. We strongly encourage code deposition in a community repository (e.g. GitHub). See the Nature Portfolio guidelines for submitting code & software for further information.

Data

Policy information about availability of data

All manuscripts must include a data availability statement. This statement should provide the following information, where applicable:

- Accession codes, unique identifiers, or web links for publicly available datasets
- A description of any restrictions on data availability
- For clinical datasets or third party data, please ensure that the statement adheres to our policy

RNA sequencing data BioProjet PRJNA1116706 ; Proteomic data PXD054068 ; Interactomic data PXD052311.
Genome of Rhipicephalus microplus is the NCBI-RefSeq: GCF_013339725.1

Research involving human participants, their data, or biological material

Policy information about studies with human participants or human data. See also policy information about sex, gender (identity/presentation), and sexual orientation and race, ethnicity and racism.

Reporting on sex and gender	N/A
Reporting on race, ethnicity, or other socially relevant groupings	N/A
Population characteristics	N/A
Recruitment	N/A
Ethics oversight	N/A

Note that full information on the approval of the study protocol must also be provided in the manuscript.

Field-specific reporting

Please select the one below that is the best fit for your research. If you are not sure, read the appropriate sections before making your selection.

☒ Life sciences ☐ Behavioural & social sciences ☐ Ecological, evolutionary & environmental sciences

For a reference copy of the document with all sections, see nature.com/documents/nr-reporting-summary-flat.pdf

Life sciences study design

All studies must disclose on these points even when the disclosure is negative.

Sample size	No sample size was calculated. A minimum of three independent measurements were performed, which is general practice and is enough to calculate statistical significance and p-values.
Data exclusions	No data were excluded from the analysis
Replication	All experiments were performed independently at least three times to ensure reproducibility. All independent replicates were successful. RNA sequencing, proteomic and interactomic was performed in 4 independent replicates.
Randomization	Cells taken from the same culture were randomly assigned as mock or infected samples.
Blinding	Not applicable due to the exploratory nature of the study. Experimental design entailed pre-defined questions.

Reporting for specific materials, systems and methods

We require information from authors about some types of materials, experimental systems and methods used in many studies. Here, indicate whether each material, system or method listed is relevant to your study. If you are not sure if a list item applies to your research, read the appropriate section before selecting a response.

Materials & experimental systems

n/a	Involved in the study
☐	☒ Antibodies
☐	☒ Eukaryotic cell lines
☒	☐ Palaeontology and archaeology
☒	☐ Animals and other organisms
☒	☐ Clinical data
☒	☐ Dual use research of concern
☒	☐ Plants

Methods

n/a	Involved in the study
☒	☐ ChIP-seq
☒	☐ Flow cytometry
☒	☐ MRI-based neuroimaging

Antibodies

Antibodies used	Rabbit anti-SFTSV N antibody (1:500) No dilution for IP<br>Mouse anti-Tubulin antibody (Sigma - T6199) use for IP no dilution<br>Goat anti-rabbit IgG (H+L) Alexa Fluor 568 (1:1000)
Validation	SFTSV N antibody were previously tested (https://doi.org/10.1128/jvi.03432-14)<br><br>Mouse anti-Tubulin antibody (Sigma-T6199) https://www.sigmaaldrich.com/GB/en/product/sigma/t6199?msockid=13a19a06638469ff18518f6f624f6816<br><br>Goat anti-rabbit IgG (H+L) Alexa Fluor 568 https://www.thermofisher.com/antibody/product/Goat-anti-Rabbit-IgG-H-L-Cross-Adsorbed-Secondary-Antibody-Polyclonal/A-11011

Eukaryotic cell lines

Policy information about cell lines and Sex and Gender in Research

Cell line source(s)	Lesley Bell-Sakyi (University of Liverpool- Tick cell Biobank) provided the tick cell line BME/CTVM6 cells derived from embryos of Rhipicephalus microplus tick. Bell-Sakyi, L., Darby, A., Baylis, M. & Makepeace, B.L. The Tick Cell Biobank: A global resource for in vitro research on ticks, other arthropods and the pathogens they transmit. Ticks Tick Borne Dis 9, 1364-1371 (2018).<br><br>VeroE6 were obtained from Michele Bouloy (Institut Pasteur, Paris). Original stock ATTC CRL-1586
Authentication	None of the cell lines were authenticated
Mycoplasma contamination	Cells were not tested for mycoplasma
Commonly misidentified lines (See ICLAC register)	NA

Plants

Seed stocks	NA
Novel plant genotypes	NA
Authentication	NA
