# Supplementary material for: Multi-omics analysis of SFTS virus infection in Rhipicephalus microplus cells reveals antiviral tick factors
Source: Nat Commun. 2025 May 21;16:4732. doi: 10.1038/s41467-025-59565-w (PMC12095547; doi:10.1038/s41467-025-59565-w)
Supplement: Supplementary file 9 — Transparent Peer Review file [file 41467_2025_59565_MOESM9_ESM.pdf]

Peer Review File

Multi-omics analysis of SFTS virus infection in Rhipicephalus microplus cells reveals antiviral tick factors

Corresponding Author: Dr Marine Petit

This file contains all reviewer reports in order by version, followed by all author rebuttals in order by version.

Version 1:

Reviewer comments:

Reviewer #1

(Remarks to the Author)
Comments: The referred manuscript evaluates the response of the R. microplus cell line during SFTSV infection. The approach includes PIT, AP-MS, and dsRNA knock-down to better understand the interaction between this pathogen and vector cells. Few clarification could improve the clarity of the scientific content.

Lines 175-176: Given the nature of the approach (transcriptomics and proteomics), could this statement be subject to overinterpretation?

Lines 204-205: Consider specifying the time point and group associated with the results for clarity.

Line 210: Consider specifying the time point for clarity.

Lines 262-263: Please consider providing a clearer explanation of the transfection validation using viral gene targets and experimentally using host targets.

Lines 265-267: Did the transfection affect cellular viability? Please add information on whether it could consequently affect the viral load.

Lines 268-269: Is the knockdown level as expected? Was additional standardization performed?

Lines 279-280: What are the effects on cell viability?

Lines 424-426: Consider adding a reference/citation or standardizing protocol.

Lines 476-478: Consider adding the reasoning for running time points differently.

Lines 549-550: Include a description of the validation of transfection and/or controls.

Line 564: Consider adding a citation for the gene reference choice.

Line 575: Consider adding a citation/reference for the anti-viral antibody.

Figure 1a: The images are too small for ideal visualization of results.

Line 672: The description of the axis does not match the graphic label.

Figure 6: This seems to be mistakenly referred as Figure 5. For Image b, clarify if the Y-axis represents fold change in relative gene expression. Is the low difference between 3-6 dpi expected? Please consider adding clarification on how the control was calculated and whether large variation in the infected group is expected.

Reviewer #2

(Remarks to the Author)
Summary:

In the manuscript, “Multi-omics analysis of severe fever with thrombocytopenia syndrome virus infection in Rhipicephalus microplus cells reveals antiviral tick factors.”, Petit et al. describe their broad multi-omics approach to characterize interactions between SFTSV and R. microplus ticks cells. Overall, this manuscript provides an interesting dataset and some interesting hypotheses to seed future studies into this virus and tick vector.

General comments:

Overall, the figures are busy, and the panel labeling is confusing. Many panels could be moved to supplemental figures.

Greater introduction or discussion regarding whole R. microplus transcriptomes and proteomes should be included, and it should be indicated why the proteome and transcriptome of cell line is necessary and an improvement.

Include statistical significance and descriptive statistics throughout the manuscript.

Specific comments:

Line 52: “Threat” should be “treat”.

Lines 95-105: The focus on SFTSV in the rationale for this section was confusing. I understand that SFTSV is the point of the manuscript overall, but not this section. Just focus on why the cell transcriptome and proteome is important.

Line 171 (and elsewhere): State p-values or other measures of significance in the text.

Lines 174-176: Please reword for clarity. The clustering was accentuated, but the positive correlation decreased.

Lines 192-194: Citation needed

Lines 195-197: This sentence is unclear.

Lines 198-199: Are these pathways significantly upregulated? This and other sections could use measures of significance and descriptive statistics in the text.

Line 201 and Figure 3: The panel labeling on this figure is confusing. Perhaps break it into smaller panels?

Section starting line 222/Figure 4: Was there significant enrichment of the highlighted groupings in the selected proteins?

Section starting 254/Figure 6: The wrong figure seems to be referenced in the text (both figure number and panels). Figure 6 should be figure 5?

Section starting 254: Are homologous proteins seen in H. longicornis or other potential SFTSV vector species?

Figure 6: The plaque assays look nice, but in several cases the RT-PCR does not reflect the plaque assay, and exhibits a high degree of variation. The lack of concurrence between these two experiments should be addressed and discussed, or RT-PCR experiments should be repeated (perhaps with new primers or increased sample size?).

Reviewer #3

(Remarks to the Author)
I co-reviewed this manuscript with one of the reviewers who provided the listed reports. This is part of the Nature Communications initiative to facilitate training in peer review and to provide appropriate recognition for Early Career Researchers who co-review manuscripts.

Reviewer #4

(Remarks to the Author)
The manuscript “Multi-omics analysis of severe fever with thrombocytopenia syndrome virus infection in Rhipicephalus microplus cells reveals antiviral tick factors” from Petit et al. uses transcriptomics and proteomics to study tick host response to SFTSV virus infection. The authors employ BME/CTVM6 cells derived from R. microplus as model system and perform a novel BME6 cell genome annotation. With the improved BME6 transcript and protein information the authors want to achieve a better understanding of SFTSV infection mechanisms in this cell system. They investigate the effects of SFTSV infection on transcriptome and proteome level at 3 and 6 days past infection and perform a comparison of affected pathways and differences between the omics layers. Further they perform an interactome analysis of the SFTSV N protein and test the effect of RNAi knockdown on SFTSV infection for several potential antiviral targets identified in the muti-omics screen.

Overall, I do support the publication of this manuscript in Nature communications, but with major revision of the gene annotation, transcriptomic and proteomic presentation section and supplementary files:
Major comments:
1) The authors write that “generation of BME6 cell de novo transcriptome and proteome” was central to the characterization of SFTSV infection. They present novel gene candidates and novel alternative splicing events in general but do not show specific improvements with regard to SFTSV infection associated genes.
In order to support this statement please state more clearly what improvement the new assembly/annotations provided in comparison to existing annotation with regard to the SFTSV infection or rephrase the sentence.

2) How many of the novel gene candidates and alternative splicing events are supported by peptide evidence from the proteomics study in this project? Please add this information to the manuscript.
How exactly is the proteomics information used in the PASA pipeline for gene prediction? To my understanding the PASA pipeline only makes use of the RNAseq data. The authors write in line 139 that “..These demonstrate the value of PIT analysis regarding the study of organisms with poor genome annotation…”. If only the transcriptomics but not the proteomics data is used directly to improve gene annotation, please rephrase this statement.

3) The authors write that they identified “29,118 genes in the BME6 transcriptomes”. Supplementary table 2 tab “D3D6_transcriptomics_and_eggnog” contains 184,585 trinity_ref entries which can be collapsed to 141,978 unique trinity_ref IDs. Which of these IDs correspond to the 29,118 transcripts?
Supplementary table 2 also lists the results for the comparative transcriptome analysis of 3dpi and 6dpi vs mock. The same TRINITY_ref entries are duplicated and in part contain different values for log2 Fold Change and padj. E.g. TRINITY_DN11890_c1_g1. Which values were used to plot Figure 2 a) and b)? With the currently provided data tables and descriptions Figure 2 a) and b) cannot be reproduced. Please provide the underlying source data.

4) Figure 1d. The numbers displayed in Fig 1d and the legend do not match. Figure total count (uncharacterized:7,363; protein search: 583; ortholog search: 1,207; blast search: 15,660; Rmic transcripts: 4,304) adds up to 29,117 transcripts. The total count should be 29,118 according to the figure title. In the figure legend the Rmic category is not listed at all, and the numbers in the remaining categories (blast search: 6,332; ortholog search: 16,683; protein search: 583; uncharacterized: 7,883) add up to a total of 31,481 transcripts.
The manuscript text (Line 124-126) states the same numbers as the Figure display for blast search (15,660) and ortholog search (1,207). Please correct this.

In the method section for “Proteomics informed by transcriptomics analysis” the authors mention “using Transdecoder software to produce ~6.8 million ORFs”. Was the DIA database search really done against 6.8 mio ORF entries or against the sequences in Supplementary_file_1 (17,527 entries)? You write that the identified ORFs were filtered for a start codon and > 66 amino acid length. Does this filter step result in the 17,527 entries in Supplementary_file_1? If not, what are the entries in Supplementary_file_1?
Please rewrite this section for clarity or provide more information on how the DIA database search was performed.

5) Figure 2. As mentioned above Figure panels a) and b) cannot be reproduced with the information in Supplementary table 2. In addition, the numbers of up- or down-regulated genes in panel a), b) and c) don’t exactly match: 3dpi correct. 6dpi - up-regulated: c) total of 579 (357+222), 528 stated in b).

The same also applies to Figure 2 panels d)-f) and Supplementary table 3. Numbers don’t match between figure, figure legend and text and the volcano plots cannot be reproduced by applying the stated filter criteria to table 3.
d,e,f) up-regulated: 3dpi f) total of 59, d) 135. 6dpi f) total of 835, e) 1238
d,e,f) down-regulated: 3dpi f) total of 7, d) 9. 6dpi f) total of 383, e) 387
The Volcano plot graphs in panel d) and e) show a curved significance cutoff instead of the stated cutoff Log2D +- 1.5, padj < 0.05. Please correct this, so the figure presentation and figure legend match each other. Please also update Supplementary table 3 “Significantly enriched target” columns to fit the cutoff specified in the manuscript text method section and Figure 2.
Currently the numbers of significant proteins after 3dpi or 6dpi in Supplementary table 3 (total entries: 4,558) do not match the Figure 2 values either (3dpi total significant: 248. Up-regulated (236), down-regulated (12); 6dpi total significant: 2,756. Up-regulated (2,281), down-regulated (475))

Supplementary table 3 lists 4,558 proteins. In the manuscript text the authors write in line 116 “.. identified 7,511 unique protein groups, with 4,750 of these groups confirmed by the presence of six or more peptides”. How are the criteria for the selection of the 4,558 proteins? Please add this information to the manuscript.

6) Please provide the source data for Figure 2 g) and h) or update Supplementary Table 2 and 3, so the information can be extracted from there. Please describe in the methods section based on which ID transcript and protein values are matched. Is this done by the “Trinity gene ID”?

Minor comments:
Line 107: customized instead of customed
Line 129: The text states “92% of the BME6 proteins identified” (Figure 1e). What does this number refer to? According to Figure 1e, Rh.microplus accounts for ~85% proteome annotation and Rh.sanguineus for 9%. Together that would make 94% of the proteome with annotation for Rhipicephalus instead of 92%.
Line 232-234: The number for interactors identified in both 3dpi and 6 dpi is given as 22 in the manuscript text and Figure 4, but 26 according to Supplementary table 4. Please correct.

Line 277-: Figure numbers 5 d1-g1 in part don't match between manuscript text and figure/figure legend. Please correct.
Line 597: Please correct the first PXD identifier (PXDXXXX).
Line 631. Figure 1d,e. Rhipicephalus is abbreviated Rh. Instead of R. Please make the abbreviation consistent between the figures, figure legends and manuscript text.
Line 664, 670, 673: (log2 FC+/- 1.5, padj < 0.5). I think this should be padj < 0.05.
Line 677. Figure 3: For convenience please provide the TRINITY Identifier, log2FC and log2D values for the genes listed in Figure 3b as additional Supplementary table or add the gene name information to the Supplementary Table 2 and 3.
Line 736. Extended Figure 2: Panel legend c)"Significant changes in protein expression" should be "transcript expression". The color code stated in the legend is wrong. Should be the same for Gene (c) and for Protein (d) panel: 3 dpi (orange), 6 dpi (purple), both (green), or none (grey).

Version 2:

Reviewer comments:

Reviewer #1

(Remarks to the Author)
The authors have incorporated the necessary revisions, which have significantly improved the clarity and depth of the study.

Reviewer #4

(Remarks to the Author)
I would like to thank the authors for adequately addressing all comments. I only have a few minor corrections for the revised manuscript.

Figure 1b/ Line 125-132: For the Trinotate, EggNog and InterPro methodology count numbers mentioned in the figure legend and manuscript text should be made congruent.
Supplementary Figure 1f. Bar plot legend IRE19 Rhabdovirus – Nucleoprotein / ORF4 / replicase without "3" at the end.
Supplementary Figure 2c. Double labeling of panels with c. No mention of panel 2c in the manuscript text.
Figure 2a,b: y-axis labeling should be "-Log10p", not "Log10p".
Figure 2f: Please check and adjust the manuscript text lines 171-173 and the numbers shown in the figure panel venn diagramm to match. According to the Figure 2f "95" proteins are significantly up-regulated at both 3 and 6 d.p.i. (text "59"). "6" proteins are down-regulated at both 3 and 6 d.p.i. (text "3"). "272" proteins are unique down-regulated in 6 d.p.i (text "88").
Line 292: Supplementary Table 5 mentioned but not included as file.

Reviewer #5

(Remarks to the Author)
No other comments or concerns here. The authors have satisfactorily responded to my review.

Open Access This Peer Review File is licensed under a Creative Commons Attribution 4.0 International License, which permits use, sharing, adaptation, distribution and reproduction in any medium or format, as long as you give appropriate credit to the original author(s) and the source, provide a link to the Creative Commons license, and indicate if changes were made.
In cases where reviewers are anonymous, credit should be given to 'Anonymous Referee' and the source.
The images or other third party material in this Peer Review File are included in the article's Creative Commons license, unless indicated otherwise in a credit line to the material. If material is not included in the article's Creative Commons license and your intended use is not permitted by statutory regulation or exceeds the permitted use, you will need to obtain permission directly from the copyright holder.
To view a copy of this license, visit https://creativecommons.org/licenses/by/4.0/

REVIEWER COMMENTS

We thank the reviewer for their very valuable commentaries and suggestions. Please see the merged document highlighting changes to the first version of the paper. Responses to reviews are indicated below.

Reviewer #1 (Remarks to the Author):

Comments: The referred manuscript evaluates the response of the R. microplus cell line during SFTSV infection. The approach includes PIT, AP-MS, and dsRNA knock-down to better understand the interaction between this pathogen and vector cells. Few clarification could improve the clarity of the scientific content.

We greatly appreciate the reviewer's thorough evaluation and insightful comments. We have carefully considered all feedback and have made substantial revisions to the manuscript in response. These modifications include:

1. Adding new supplementary figure panels to provide additional data and clarity
2. Refining the main text to enhance precision and address specific concerns
3. Incorporating additional analyses where necessary

Detailed responses to each comment, along with the corresponding modifications, are provided below. We believe these changes have significantly strengthened the manuscript and addressed all the reviewer's concerns.

Lines 175-176: Given the nature of the approach (transcriptomics and proteomics), could this statement be subject to overinterpretation?
We changed sentence to add more nuance to our statement. "*suggesting that SFTSV infection may impact either post-transcriptional or translational processes in BME6 cells*."

Lines 204-205: Consider specifying the time point and group associated with the results for clarity.
Line 210: Consider specifying the time point for clarity.
We added throughout the text more details regarding the time points and groups associated. See the following example: " *AP-1 complex transcription factor part of the JNK pathway (log2D= 2.3 (p-value=0.06) and log2D=4.5 (p-value=0.004) at 3 and 6 d.p.i. respectively)*"

Lines 262-263: Please consider providing a clearer explanation of the transfection validation using viral gene targets and experimentally using host targets.

We further explained our rationale behind our selection of SFTSV N segment silencing for KD validation: "To evaluate the effectiveness of our approach, we first tested it on SFTSV. We hypothesized that successful dsRNA-mediated knock-down would reduce viral RNA levels, leading to decreased viral mRNA and genome production and consequently viral titers."

Lines 265-267: Did the transfection affect cellular viability? Please add information on whether it could consequently affect the viral load. **Lines 279-280:** What are the effects on cell viability?

To address the reviewer's concern about cell viability, we conducted a comprehensive assessment using the CellTiter-Glo® Luminescent Cell Viability Assay (Promega). This assay quantifies cellular ATP levels, providing a direct measure of metabolically active cells. BME-CTVM6 cells were transfected with dsRNA targeting our genes of interest, including SFTSV

N, and a luciferase control. Cell viability was measured at 3 and 6 days post-transfection. The results are presented in Supplementary Figure 6e. Statistical analysis (Two-way ANOVA) revealed no significant differences in cell viability between the targeted gene knockdowns and the negative control (dsRNA targeting *Renilla* luciferase). This demonstrates that neither the transfection process nor the knockdown of our selected targets significantly impacted BME-CTVM6 cell viability significantly. These findings suggest that any observed changes in viral load are due to the specific gene knockdowns rather than altered cell viability, validating the robustness of our experimental approach.

Lines 268-269: Is the knockdown level as expected? Was additional standardization performed?

This publication presents for the first time the dsRNA mediated silencing of SFTSV S segment in tick cells, so no "expected" level as such can be referred to. When comparing to the available literature, for example Barry and al 2012 which tested dsRNA-mediated silencing of SFV in various tick cells. They show a 1 log decrease when measuring with luciferase in *Rhipicephalus microplus* BME/CTVM23 cells, like our observation for the plaque assay. Confirming our observations that *Rhipicephalus* tick have a working RNAi pathway.

Lines 424-426: Consider adding a reference/citation or standardizing protocol.
Our protocol was adapted from Fauver et al. 2019 as mentioned in the text line 468 reference 29 (also mentioned in the result section).

Lines 476-478: Consider adding the reasoning for running time points differently. The PIT experiment was initially designed to test protein expression at 3 d.p.i. However, we observed minimal changes in protein expression at this time point. Considering the slow metabolism and protein turnover rates in embryo-derived BME/CTVM6 cells, we decided to extend our analysis to a later time point of 6 d.p.i. This extension allowed for additional viral replication cycles in the infected tick cells, potentially revealing more significant changes in protein expression.

Lines 549-550: Include a description of the validation of transfection and/or controls.

We added to the method for dsRNA production and transfection, and the control we used. "For control fluorescein (Fl)-dsRNA we replaced dUTP by dUTP-Fl. […] Control for efficient transfection were performed with fluorescein labelled dsRNA targeting Renilla luciferase"

Line 564: Consider adding a citation for the gene reference choice.
We added references used for our choice of the RSP4 gene of reference in the text. See line 640: "RPS4 RNA[98,99]"

Line 575: Consider adding a citation/reference for the anti-viral antibody.
The anti-viral antibody has been produced following the protocol described in Brennan et al. 2015 (J virol). This reference is listed in the main text and the method section. "with the primary antibody, rabbit anti-SFTSV N[81]".

Figure 1a: The images are too small for ideal visualization of results.

We enlarged the pictures and zoomed in for better visualization of infected and non-infected cells. Panel is now located in Supplementary Figure 1a.

Line 672: The description of the axis does not match the graphic label.

We modified the axis titre to match the graph label.

Figure 6: This seems to be mistakenly referred as Figure 5. For Image b, clarify if the Y-axis represents fold change in relative gene expression. Is the low difference between 3-6 dpi expected? Please consider adding clarification on how the control was calculated and whether large variation in the infected group is expected.
We added to the Y-axis the relative gene expression, and we also specified the gene of reference we used. This was done for panels b and c.

Reviewer #2 (Remarks to the Author):

Summary:

In the manuscript, "Multi-omics analysis of severe fever with thrombocytopenia syndrome virus infection in Rhipicephalus microplus cells reveals antiviral tick factors.", Petit et al. describe their broad multi-omics approach to characterize interactions between SFTSV and R. microplus ticks cells. Overall, this manuscript provides an interesting dataset and some interesting hypotheses to seed future studies into this virus and tick vector.

General comments:

Overall, the figures are busy, and the panel labeling is confusing. Many panels could be moved to supplemental figures.
We changed our figure design and moved some panel to supplementary figures. Figure 1-3-4-5 have all been modified for more clarity. We now have 7 Supplementary Figures, instead of 3 extended figures.

Greater introduction or discussion regarding whole R. microplus transcriptomes and proteomes should be included, and it should be indicated why the proteome and transcriptome of cell line is necessary and an improvement.

We added lines in the Results section to explain the importance of PIT for BME6 cells.
See below: "In the absence of a well-annotated genome for *R. microplus*, we employed a Proteomics informed by Transcriptomics (PIT) approach (Fig. 1a), which integrates transcriptomic data to create high-quality protein search databases. This approach is particularly advantageous for non-model organisms with incomplete genomic resources, as it enables the validation of predicted transcripts, identification of novel open reading frames (ORFs), and characterization of previously unreported splicing events. By combining transcriptomic and proteomic datasets, PIT provides enhanced resolution of cellular pathways and processes, offering new insights into tick-specific biology, and characterization of host-pathogens interactions."

Include statistical significance and descriptive statistics throughout the manuscript.
We added values and statistics throughout the manuscripts.

Specific comments:

Line 52: “Threat” should be “treat”.
This has been corrected.

Lines 95-105: The focus on SFTSV in the rationale for this section was confusing. I understand that SFTSV is the point of the manuscript overall, but not this section. Just focus on why the cell transcriptome and proteome is important.

We detailed the importance of PIT for the study of BME6 cells derived from *R. microplus*. See line 91-99: “In the absence of a well-annotated genome for *R. microplus*, we employed a Proteomics informed by Transcriptomics (PIT) approach (Fig. 1a), which integrates transcriptomic data to create high-quality protein search databases. This approach is particularly advantageous for non-model organisms with incomplete genomic resources, as it enables the validation of predicted transcripts, identification of novel open reading frames (ORFs), and characterization of previously unreported splicing events. By combining transcriptomic and proteomic datasets, PIT provides enhanced resolution of cellular pathways and processes, offering new insights into tick-specific biology, and characterization of host-pathogens interactions.”

Line 171 (and elsewhere): State p-values or other measures of significance in the text.
We apologise for this omission; p-values and differential gene expression values have been added throughout the text.

Lines 174-176: Please reword for clarity. The clustering was accentuated, but the positive correlation decreased. This was amended in the text.

Lines 192-194: Citation needed.

We added an additional reference in the text. This new study used comparative genomics to annotate the *R. microplus* genome. They observe that 66% of the genome do not have orthologs and therefore could not be associated with GO category (reference Wang et al. 2007). This underscore the lack of annotation and proper GO category for *R. microplus*.

Lines 195-197: This sentence is unclear.

We have re-written the sentence to improve clarity : “To elucidate the comprehensive antiviral response mechanisms against SFTSV infection and delineate the core immune pathways in BME6 cells, we integrated previously identified tick-derived immune effectors[20, 23, 47] with immune effectors identified in our annotated proteome”.

Lines 198-199: Are these pathways significantly upregulated? This and other sections could use measures of significance and descriptive statistics in the text.

No GO category for IMD, Jak-STAT or IMD is available, so we are unable to conclude on the regulation of those pathway globally. We tried running additional pathway analysis using DAVID software but no GO related to “response to infection”, “innate immune pathway” were identified, highlighting the lack of GO category for this group in *R. microplus* annotation. We therefore just assessed identified effectors.

Line 201 and Figure 3: The panel labelling on this figure is confusing. Perhaps break it into smaller panels?

We did re-arrange the figure. Each innate immune pathway was separated in panels. Panel 3a for the Toll pathway, 3b for Imd and JNK and 3c for JAK-STAT pathways. The remaining genes were separated in 2 other panels: 1) antiviral effectors (3d) and 2) stress-related pathways (3e).

We believe this makes the panel clearer.

Section starting line 222/Figure 4: Was there significant enrichment of the highlighted groupings in the selected proteins?

We tested interactome enrichment using Cytoscape Tools enrichment, no enrichment when term like *Rhipicephalus microplus*, or *Ixodes scapularis* were tested. When trying *Homo sapiens*, most enriched category were perichromatin fibrils, and cytosol, the rest was related to RNA transport, export. All those information was unrelated to SFTSV infection in tick cells. So we decided to highlight in Figure 4 groups of proteins which were concordant with our observation for both transcriptomics and proteomics analyses.

Section starting 254/Figure 6: The wrong figure seems to be referenced in the text (both figure number and panels). Figure 6 should be figure 5?

We replaced all wrong mentions of Figure 6 in the text.

Section starting 254: Are homologous proteins seen in H. longicornis or other potential SFTSV vector species?

To screen for orthologs (homologous proteins) we ran a BLASTp search for the 9 selected target in our biological validation (HSP68, Microplusin, Croquemort, SCARB1, TRAF2, DHX9, PAPB4, SMG7, UPF1) against *Haemaphysalis longicornis*. Homologs targets were found for 7 out of 9 proteins. We provided the coverage and identity percentage for all and the gene ID for corresponding *H. longicornis* in the Supplementary Figure 7.

Figure 6: The plaque assays look nice, but in several cases the RT-PCR does not reflect the plaque assay and exhibits a high degree of variation. The lack of concurrence between these two experiments should be addressed and discussed, or RT-PCR experiments should be repeated (perhaps with new primers or increased sample size?).

To assess the reviewer's point we used a new house-keeping gene RPS4, which show less variability across mock and infected samples (SFTSV/Mock Ct variations observed: ±1Ct). We used our cDNAs to re-run the qPCR. Most targets did not exhibit major differences, but we improved our read out for RT-qPCR of SFTSV M segment reducing some of the high variations observed previously. However, we still observed differences between the mRNA level and the viral titers, and we provided the following explanation (line 329-342):

"To assess the impact of target gene silencing on viral RNA levels in SFTSV-infected cells (MOI 0.5 PFU/ml), we quantified the viral M segment by RT-qPCR. At 3 d.p.i., only knockdown of SMG7 and UPF1 resulted in significantly lower M segment levels. These differences were not observed at 6 d.p.i. At this later timepoint, dsRNA knockdown of UPF1 resulted in increased viral RNA levels (Fig. 5d2). Other genes, including Hsp68, Croquemort,

and DHX9, exhibited higher expression of the SFTSV M segment at 6 d.p.i. However, minor variations in values affected statistical significance. These observed variations of fold change at 6 d.p.i. could result of knock-down efficiency variations, or represent the inherent heterogeneity of the BME6 cells, which are derived from embryonic tick tissues28.

While RNA quantification can indicate changes in intracellular viral replication levels, it does not necessarily correlate with the production and release of infectious virus particles. Therefore, to obtain a more accurate measure of infectious virus production, we determined viral titre in the supernatant of knockdown (KD) cells by plaque assay (Fig. 5a3-d3), thereby measuring the impact of the gene KD on viral infectivity..”

Reviewer #3 (Remarks to the Author):

I co-reviewed this manuscript with one of the reviewers who provided the listed reports. This is part of the Nature Communications initiative to facilitate training in peer review and to provide appropriate recognition for Early Career Researchers who co-review manuscripts.

Reviewer #4 (Remarks to the Author):

The manuscript “Multi-omics analysis of severe fever with thrombocytopenia syndrome virus infection in Rhipicephalus microplus cells reveals antiviral tick factors” from Petit et al. uses transcriptomics and proteomics to study tick host response to SFTSV virus infection. The authors employ BME/CTVM6 cells derived from R. microplus as model system and perform a novel BME6 cell genome annotation. With the improved BME6 transcript and protein information the authors want to achieve a better understanding of SFTSV infection mechanisms in this cell system. They investigate the effects of SFTSV infection on transcriptome and proteome level at 3 and 6 days past infection and perform a comparison of affected pathways and differences between the omics layers. Further they perform an interactome analysis of the SFTSV N protein and test the effect of RNAi knockdown on SFTSV infection for several potential antiviral targets identified in the muti-omics screen. Overall, I do support the publication of this manuscript in Nature communications, but with major revision of the gene annotation, transcriptomic and proteomic presentation section and supplementary files:
Major comments:
1) The authors write that “generation of BME6 cell de novo transcriptome and proteome” was central to the characterization of SFTSV infection. They present novel gene candidates and novel alternative splicing events in general but do not show specific improvements with regard to SFTSV infection associated genes.
In order to support this statement please state more clearly what improvement the new assembly/annotations provided in comparison to existing annotation with regard to the SFTSV infection or rephrase the sentence.

We thank the reviewer for their insightful observations. We recognize the importance of providing greater clarity on the significance of our *de novo* transcriptome and proteome assembly in the context of SFTSV infection. To address this, we have thoroughly revised Figure 1 to clearly illustrate the specific advancements offered by our approach compared to existing annotations.

Our refined analysis integrates genome-aligned peptides with RNA-seq data, facilitating the identification of novel gene candidates and alternative splicing events directly associated with SFTSV infection. Specifically, we uncovered a total of 1,248 coding sequences (CDSs)

containing novel splicing events, among which 459 demonstrated significant differential expression during SFTSV infection (Supplementary Figure 2c). These discoveries, which were not detectable using conventional annotations, provide critical new insights into the molecular response of host cells to SFTSV and underscore the enhanced resolution and utility of our methodological approach.

2) How many of the novel gene candidates and alternative splicing events are supported by peptide evidence from the proteomics study in this project? Please add this information to the manuscript.

This was illustrated in Figure 1e.

How exactly is the proteomics information used in the PASA pipeline for gene prediction? To my understanding the PASA pipeline only makes use of the RNAseq data. The authors write in line 139 that "..These demonstrate the value of PIT analysis regarding the study of organisms with poor genome annotation…". If only the transcriptomics but not the proteomics data is used directly to improve gene annotation, please rephrase this statement.

To enhance the accuracy of our gene predictions, we integrated our proteomics datasets into the EVidenceModeler (EVM) pipeline. Specifically, we employed the exonerate function protein2genome to align our experimentally derived peptides to the genome. These peptide-genome alignments were then provided as additional evidence to the EVM program, significantly improving the precision of gene structure predictions.
This has been added for clarity in the text (line 137-141):

"Finally, we opted to advance our genome annotation efforts by predicting alternative splicing events. To achieve this, we assembled Trinity-derived contigs using the Program to Assemble Spliced Alignments (PASA) software[40], followed by validation with the EVIdenceModeler (EVM) program[41], which integrates multiple sources of evidence such as PASA transcripts, protein alignments, and gene prediction tools like Augustus[42]."

And in the Methods section (line 547-549):

"Our PASA RNA-seq gene expression results were combined using EVidenceModeler to ab initio gene prediction (Augustus v3.5.0[84], SNAP[85]) and protein alignments (exonerate v2.4.0[86], miniprot v0.12[87], genome threader[88])."

3) The authors write that they identified "29,118 genes in the BME6 transcriptomes". Supplementary table 2 tab "D3D6_transcriptomics_and_eggnog" contains 184,585 trinity_ref entries which can be collapsed to 141,978 unique trinity_ref IDs. Which of these IDs correspond to the 29,118 transcripts?

The previously mentioned 29,118 genes was an error. This number actually refers to the annotations generated by the PASA-EVM software, not the total gene count in the BME6 transcriptomes. We have modified Supplementary Table 2 to focus on Trinity contig annotations. The table now includes: i) Trinity contig information; ii) Ortholog data; iii) Protein domain annotations; iv) Reference PASA coding sequences (asmbl IDs). Furthermore we have now provided the dataset used for Figure 2 in the source data, enhancing transparency and reproducibility.

Supplementary table 2 also lists the results for the comparative transcriptome analysis of 3dpi and 6dpi vs mock. The same TRINITY_ref entries are duplicated and in part contain different values for log2 Fold Change and padj. E.g. TRINITY_DN11890_c1_g1. Which

values were used to plot Figure 2 a) and b)? With the currently provided data tables and descriptions Figure 2 a) and b) cannot be reproduced. Please provide the underlying source data.

We apologies for the confusion link to our Supplementary Table 2. In our volcano plots all Trinity_ID were plots, to avoid confusion we used asmbl ID (cDNA contigs made by PASA). All the data to reconstruct the Volcano plots is now provided as source data, and our supplementary table 2 focuses on the functional annotation of the transcriptomes, which encompasses both Trinity isoforms, Trininty genes and asmbl ID.

4) Figure 1d. The numbers displayed in Fig 1d and the legend do not match. Figure total count (uncharacterized:7,363; protein search: 583; ortholog search: 1,207; blast search: 15,660; Rmic transcripts: 4,304) adds up to 29,117 transcripts. The total count should be 29,118 according to the figure title. In the figure legend the Rmic category is not listed at all, and the numbers in the remaining categories (blast search: 6,332; ortholog search: 16,683; protein search: 583; uncharacterized: 7,883) add up to a total of 31,481 transcripts.
The manuscript text (Line 124-126) states the same numbers as the Figure display for blast search (15,660) and ortholog search (1,207). Please correct this.
All numbers have been checked and corrected in Figures, Figure legends and in the main text.

In the method section for “Proteomics informed by transcriptomics analysis” the authors mention “using Transdecoder software to produce ~6.8 million ORFs”. Was the DIA database search really done against 6.8 million ORF entries or against the sequences in Supplementary_file_1 (17,527 entries)? You write that the identified ORFs were filtered for a start codon and > 66 amino acid length. Does this filter step result in the 17,527 entries in Supplementary_file_1? If not, what are the entries in Supplementary_file_1?
Please rewrite this section for clarity or provide more information on how the DIA database search was performed.

The file used for the DIA search was the trinity_out_66.fa which contains the ORF predictions. This file contains 6,885,654 entries. The Supplementary Dataset 1 with 17,527 entries was build following the initial mass-spectrometry analysis. This subset was created by selecting ORFs that matched at least one peptide in any of the analyzed samples. This curated list, presented in Supplementary Dataset 1, represents our "de novo proteome" and served as the reference for analyzing mass spectrometry results in our interactomics studies.

5) Figure 2. As mentioned above Figure panels a) and b) cannot be reproduced with the information in Supplementary table 2. In addition, the numbers of up- or down-regulated genes in panel a), b) and c) don’t exactly match: 3dpi correct. 6dpi - up-regulated: c) total of 579 (357+222), 528 stated in b).

We made a mistake in the Venn diagram as the correct total number for up-regulated (log2FC>1.5 and padj <0.05) is 528 as mentioned in the volcano plots. Venn-diagram number has been updated.

The same also applies to Figure 2 panels d)-f) and Supplementary table 3. Numbers don’t match between figure, figure legend and text and the volcano plots cannot be reproduced by applying the stated filter criteria to table 3. d,e,f) up-regulated: 3dpi f) total of 59, d) 135. 6dpi

f) total of 835, e) 1238
d,e,f) down-regulated: 3dpi f) total of 7, d) 9. 6dpi f) total of 383, e) 387
The Volcano plot graphs in panel d) and e) show a curved significance cutoff instead of the stated cutoff Log2D +- 1.5, padj < 0.05. Please correct this, so the figure presentation and figure legend match each other. Please also update Supplementary table 3 "Significantly enriched target" columns to fit the cutoff specified in the manuscript text method section and Figure 2. Currently the numbers of significant proteins after 3dpi or 6dpi in Supplementary table 3 (total entries: 4,558) do not match the Figure 2 values either (3dpi total significant: 248. Up-regulated (236), down-regulated (12); 6dpi total significant: 2,756. Up-regulated (2,281), down-regulated (475))

We updated the legend of the Figure 2 regarding the proteomics Volcano plots describing Perseus statistical analysis of significance. Line (735-738): "The y axis shows non zero-confidence for each protein (-log10(p)). Blue dots, significantly up-regulated proteins, and red dots, down-regulated proteins, were identified by using a modified t-test (Perseus "one sample t-test"; s0=0.02; Benjamini-Hochberg FDR < 0.05). Gray dots represent non-significantly regulated proteins)." We also updated the Supplementary Table 3 and provided source data for the reproduction of the Volcano Plots.

Supplementary table 3 lists 4,558 proteins. In the manuscript text the authors write in line 116 ".. identified 7,511 unique protein groups, with 4,750 of these groups confirmed by the presence of six or more peptides". How are the criteria for the selection of the 4,558 proteins? Please add this information to the manuscript.

Thanks you for this observation. We decided to focus on unique proteins; 5616 were identified and within this list 4558 had 6 peptides in at least 4 samples across our tested conditions (mock, 3dpi, or 6 dpi). This information was added line 119: "by the presence of six or more peptides for 4 analysed samples across all conditions".

6) Please provide the source data for Figure 2 g) and h) or update Supplementary Table 2 and 3, so the information can be extracted from there. Please describe in the methods section based on which ID transcript and protein values are matched. Is this done by the "Trinity gene ID"?

We used the asmbl ID for merging transcriotomics and proteomics data. Source data are now provided for the Figure 2 g) and h) graph.

Minor comments:
Line 107: customized instead of customed – This was modified
Line 129: The text states "92% of the BME6 proteins identified" (Figure 1e). What does this number refer to? According to Figure 1e, Rh.microplus accounts for ~85% proteome annotation and Rh.sanguineus for 9%. Together that would make 94% of the proteome with annotation for Rhipicephalus instead of 92%.

We changed the text for 94%.

Line 232-234: The number for interactors identified in both 3dpi and 6 dpi is given as 22 in the manuscript text and Figure 4, but 26 according to Supplementary table 4. Please correct.

We excluded these 4 interactors from the interactome presented in Figure 4, as they are related to the IRE19-rhabdovirus identified in our BME/CTVM6 cells. This decision was

made to keep the main figure focused on the core cellular interactions relevant to our study's primary aims. However, to ensure comprehensive data reporting, we have retained these interactors in the Supplementary Table. This approach allows interested readers to access the complete dataset, including these additional interactions.

Line 277-: Figure numbers 5 d1-g1 in part don't match between manuscript text and figure/figure legend. Please correct.

This was corrected in the result and discussion part of our text.

Line 597: Please correct the first PXD identifier (PXDXXXX).

We added the PXD identifier for the DIA proteomics data. Line (667): "PXD054068 and PXD052311 Source data are provided"

Line 631. Figure 1d,e. Rhipicephalus is abbreviated Rh. Instead of R. Please make the abbreviation consistent between the figures, figure legends and m

anuscript text.

This was amended in our Figure legends and revised manuscript text.

Line 664, 670, 673: (log2 FC+/- 1.5, padj < 0.5). I think this should be padj < 0.05.

This was amended in Figure legends.

Line 677. Figure 3: For convenience please provide the TRINITY Identifier, log2FC and log2D values for the genes listed in Figure 3b as additional Supplementary table or add the gene name information to the Supplementary Table 2 and 3.

We generated a new Supplementary Table connected to the Figure 3 to match the gene names and the TRINITY Identifiers. This is now Supplementary Table 4.

Line 736. Extended Figure 2: Panel legend c)"Significant changes in protein expression" should be "transcript expression". The color code stated in the legend is wrong. Should be the same for Gene (c) and for Protein (d) panel: 3 dpi (orange), 6 dpi (purple), both (green), or none (grey).

This was amended according to the reviewer comments.

Response to reviewers

We thank all reviewers for their evaluation of the revised manuscript and their minor comments. We have addressed all points raised—please see details below.

Reviewer 4:

Figure 1b/ Line 125-132: For the Trinotate, EggNog and InterPro methodology count numbers mentioned in the figure legend and manuscript text should be made congruent.

The numbers have been corrected and are now consistent across the figure, the legend, and the manuscript text.

Supplementary Figure 1f. Bar plot legend IRE19 Rhabdovirus – Nucleoprotein / ORF4 / replicase without “3” at the end.

Thank you for pointing out the typos. These have been corrected.

Supplementary Figure 2c. Double labeling of panels with c. No mention of panel 2c in the manuscript text.

We have removed the duplicate panel label. Supplementary Figure 2c is now referenced in the manuscript (line 149):
“Moreover, these annotations provided valuable insights into SFTSV infection (Supplementary Fig. 2c).”

Figure 2a,b: y-axis labeling should be “-Log10p”, not “Log10p”.

This has been corrected accordingly.

Figure 2f: Please check and adjust the manuscript text lines 171-173 and the numbers shown in the figure panel venn diagramm to match. According to the Figure 2f “95” proteins are significantly up-regulated at both 3 and 6 d.p.i. (text “59”). “6” proteins are down-regulated at both 3 and 6 d.p.i. (text “3”). “272” proteins are unique down-regulated in 6 d.p.i (text “88”).

These discrepancies have been corrected. The numbers in the figure and the manuscript text are now consistent.

Line 292: Supplementary Table 5 mentioned but not included as file.

Supplementary Table 5 has now been included and is available as Supplementary Data 6.
